# Copper-catalysed asymmetric allylic alkylation of alkylzirconocenes to racemic 3,6-dihydro-2*H*-pyrans

**DOI:** 10.3762/bjoc.11.264

**Published:** 2015-12-03

**Authors:** Emeline Rideau, Stephen P Fletcher

**Affiliations:** 1 Department of Chemistry, Chemistry Research Laboratory, University of Oxford, 12 Mansfield Road, Oxford, OX1 3TA, UK, Fax: +44(1865)285002

**Keywords:** allylic alkylation, asymmetric catalysis, copper, design of experiments, dynamic kinetic asymmetric transformation, heterocycles, Schwartz reagent

## Abstract

Asymmetric allylic alkylation is a powerful reaction that allows the enantioselective formation of C–C bonds. Here we describe the asymmetric alkylation of alkylzirconium species to racemic 3,6-dihydro-2*H*-pyrans. Two systems were examined: 3-chloro-3,6-dihydro-2*H*-pyran using linear optimization (45–93% ee, up to 33% yield, 5 examples) and 3,6-dihydro-2*H*-pyran-3-yl diethyl phosphate with the assistance of a design of experiments statistical approach (83% ee, 12% yield). ^1^H NMR spectroscopy was used to gain insight into the reaction mechanisms.

## Introduction

**Scheme 1:**
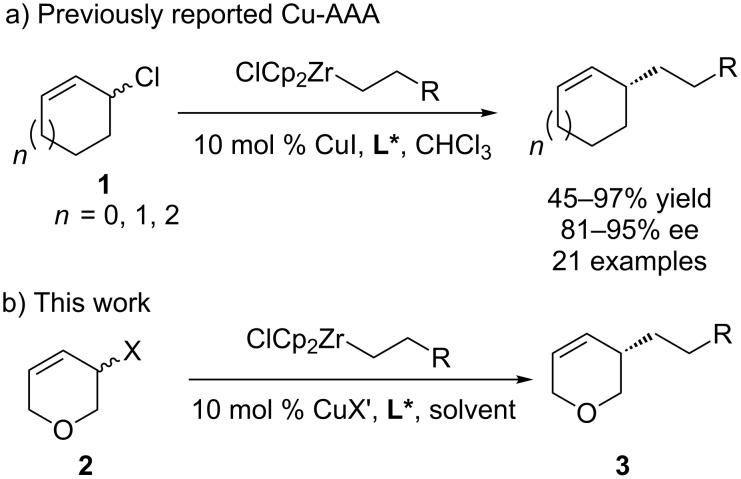
Previously reported Cu-AAA of alkylzirconium reagents to racemic allyl chlorides [26] and this work.

Asymmetry is found in many natural products and biologically active molecules. Using racemic starting materials to synthesize enantiomerically enriched products is a powerful and underdeveloped strategy [1–4]. In some cases transition metal-catalysed asymmetric allylic alkylation (AAA) reactions [5–7] can be used in dynamic kinetic asymmetric transformations (DYKAT) [8–15] to provide single enantiomer products from racemic starting materials. Mechanistically some of these have been shown to occur by direct enantio-convergent transformations [16–18]. We have developed Cu-catalysed asymmetric conjugate additions of alkylzirconium reagents generated in situ by hydrometallation of terminal alkenes [19–25], and recently demonstrated that zirconium nucleophiles may undergo highly enantioselective copper-catalysed AAAs to racemic cyclic allyl halides, such as **1** (Scheme 1a) [26–27].

Tetrahydropyrans are a common motif in natural products and pharmaceuticals and are useful synthetic intermediates. However, the direct asymmetric derivatization of pyrans is rare [28] and enantiomerically enriched tetrahydropyrans are often obtained by ring-closing methods [29–30]. To extend our previously reported DYKATs beyond all-carbon electrophiles we decided to examine 3-chloro-3,6-dihydro-2*H*-pyran (**2a**, Scheme 1b). This was envisaged to be a challenging substrate. The presence of oxygen in the ring would modify the electronics, and likely the reactivity, of the starting material. The oxygen lone pairs on **2a** could potentially interact with the copper-catalyst or alkyl metal nucleophiles.

## Results and Discussion

We first examined the in situ hydrometallation/AAA of 4-phenyl-1-butene (**4**) to racemic 3-chloro-3,6-dihydro-2*H*-pyran (**2a**, Table 1). Interestingly, no product was formed using our previously reported conditions for AAA to racemic carbocyclic substrates (CuI, ligand **A**, CHCl_3_, Table 1, entry 1) [26] and only unreacted starting material was recovered. Different Cu salts were examined (Table 1, entries 2–7) and more strongly electron withdrawing counterions were found to provide the desired product, with CuClO_4_ giving the best ee (70% ee, Table 1, entry 3). A solvent screen lead us to the conclusion that chlorinated solvents are best (CH_2_Cl_2_ (70% ee) and CHCl_3_ (67% ee), Table 1, entries 7 and 10, respectively). Extensive examination of phosphoramidite ligands (for example, Table 1, entries 2 and 11–13) did not improve the ee. We then tested many different additives (TMSCl, AgOTf, borates, ZrCl_4_, Si(OEt)_4_, etc, for example Table 1, entries 14–18). Using B(OiPr)_3_, which presumably acts as a Lewis acid, improved the ee to 80% (Table 1, entry 18) and so we re-examined different ligands using CuClO_4_ in CH_2_Cl_2_ with B(OiPr)_3_ (Table 1, entries 19–21). Derivatives of ligand **B** were tested and ligand **F** gave 83% ee (Table 1, entry 21), while electronically similar **E** was much less selective (47% ee, Table 1, entry 20). The effects of concentration, temperature and catalyst loading were also investigated (not shown) with no improvement on the enantioselectivity.

**Table 1:** Asymmetric alkylation to 3-chloro-3,6-dihydro-2*H*-pyran (**2a**)^a^.

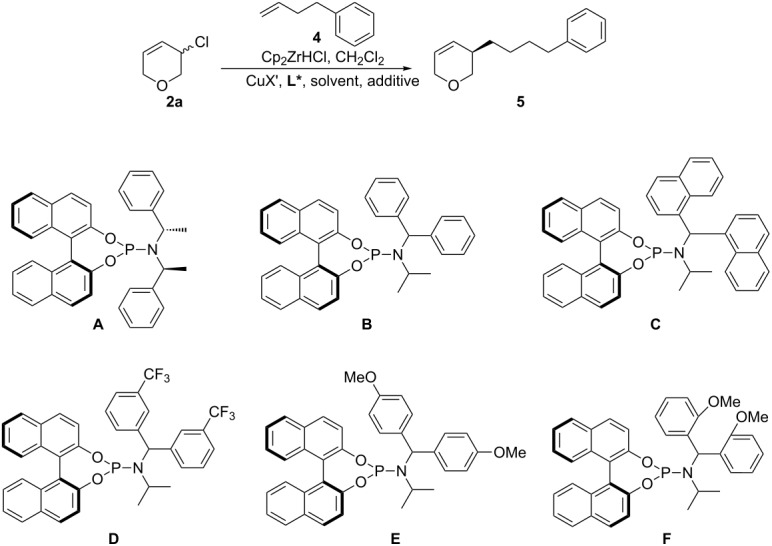					

Entry	Copper	L*	Solvent	Additive	ee^b^

1	CuI	**A**	CHCl_3_		NP
2	CuClO_4_	**A**	CH_2_Cl_2_		68%
3	CuClO_4_	**B**	CH_2_Cl_2_		70%
4	CuOTf	**B**	CH_2_Cl_2_		64%
5	CuNTf_2_	**B**	CH_2_Cl_2_		52%
6	CuTC	**B**	CH_2_Cl_2_		12%
7	CuSbF_6_	**B**	CH_2_Cl_2_		NP
8	CuClO_4_	**B**	Et_2_O		55%
9	CuClO_4_	**B**	Me-THF		38%
10	CuClO_4_	**B**	CHCl_3_		67%
11	CuClO_4_	**C**	CH_2_Cl_2_		53%
12	CuClO_4_	**D**	CH_2_Cl_2_		36%
13	CuClO_4_	**E**	CH_2_Cl_2_		12%
14	CuClO_4_	**B**	CH_2_Cl_2_	TMSCl	73%
15	CuClO_4_	**B**	CH_2_Cl_2_	Si(OEt)_4_	63%
16	CuClO_4_	**B**	CH_2_Cl_2_	Ti(OiPr)_4_	25%
17	CuClO_4_	**B**	CH_2_Cl_2_	AlCl_3_	15%
18	CuClO_4_	**B**	CH_2_Cl_2_	B(OiPr)_3_	80%
19	CuClO_4_	**C**	CH_2_Cl_2_	B(OiPr)_3_	78%
20	CuClO_4_	**E**	CH_2_Cl_2_	B(OiPr)_3_	47%
21	CuClO_4_	**F**	CH_2_Cl_2_	B(OiPr)_3_	83%

^a^Conditions: 4-phenyl-1-butene (2.5 equiv), Cp_2_ZrHCl (2.0 equiv), **2a** (1.0 equiv), CuL* as specified (0.1 equiv), additive as specified (1.0 equiv), in specified solvent (2.0 mL), room temperature. ^b^ee determined by HPLC. NP = no product. For more information on procedures see Supporting Information File 1.

After extensive optimization, the highest enantiomeric excess obtained was only 83% ee and so we decided to examine other leaving groups (Table 2). Like allyl chloride **2a**, allyl bromide **2b** gave no desired product when using our previously reported conditions [26] (Table 2, entry 2). The use of **2b** also only gave low ee when using the conditions optimized above (38% ee, Table 2, entry 1). Allyl acetate **2c** did not give the desired product under any conditions examined, however, allyl phosphate **2d** was found to provide **5** with good selectivity (77% ee, Table 2, entry 5). 3,6-Dihydro-2*H*-pyran-3-yl diethyl phosphate (**2d**) was also the only substrate to react using our previously reported AAA conditions (CuI, ligand **A**, CHCl_3_) [26], albeit with poor enantioselectivity (29% ee, Table 2, entry 6).

**Table 2:** Effect of leaving groups.^a^

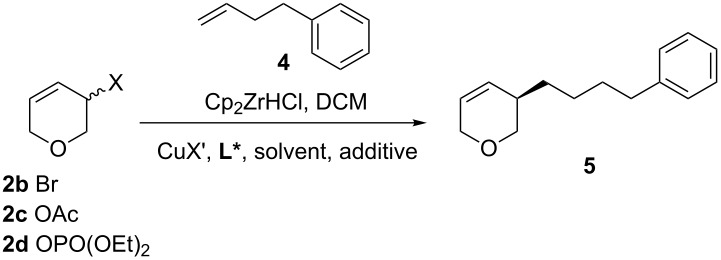						

Entry	X	Copper	L*	Solvent	Additive	ee^b^

1	Br	CuClO_4_	**F**	CH_2_Cl_2_	B(O*i*Pr)_3_	38%
2	Br	CuI	**A**	CHCl_3_		NP
3	OAc	CuClO_4_	**F**	CH_2_Cl_2_	B(O*i*Pr)_3_	NP
4	OAc	CuI	**A**	CHCl_3_		NP
5	OPO(OEt)_2_	CuClO_4_	**F**	CH_2_Cl_2_	B(O*i*Pr)_3_	77%
6	OPO(OEt)_2_	CuI	**A**	CHCl_3_		29%

^a^Conditions: 4-phenyl-1-butene (2.5 equiv), Cp_2_ZrHCl (2.0 equiv), **2** (1.0 equiv), CuL*(0.1 equiv), additive (1.0 equiv), in solvent (2.0 mL), room temperature. ^b^ee determined by HPLC. NP = no product. For more information on procedures see Supporting Information File 1.

Design of experiments (DoE) [31–37] is a powerful tool for efficient screening and is commonly used in industry, since traditional one-factor-at-a-time optimization poorly covers the available parameter space and may not locate the most optimal conditions. As DoE rapidly explores the response space efficiently and can reveal interdependence of factors at no extra experimental cost, we decided to briefly examine DoE in this complex asymmetric transformation. We note that there are important limits to this investigation. Understanding what interactions give rise to asymmetric induction (particularly in transformations where mechanisms are not understood) is extremely challenging, and it is not obvious how to parameterize the multiple variables present in key factors such as ligand structure [38].

**Figure 1:**
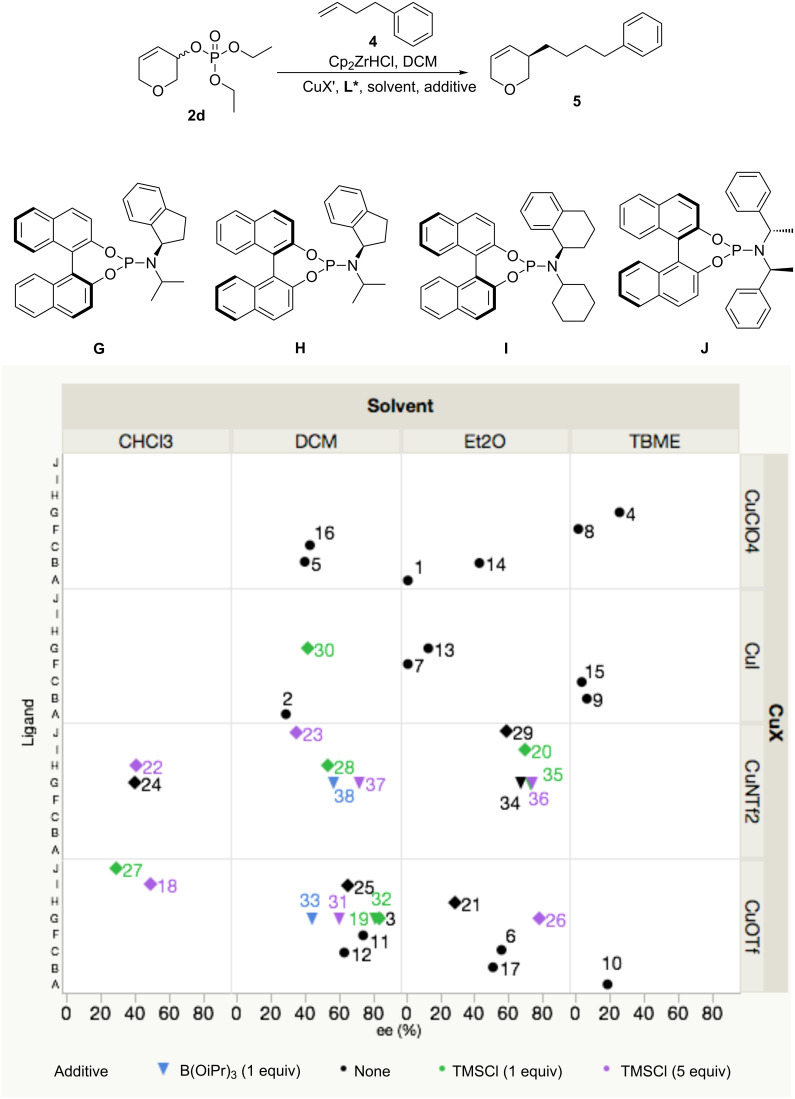
DoE from 3,6-dihydro-2*H*-pyran-3-yl diethyl phosphate (**2d**). Conditions: 4-phenyl-1-butene (2.5 equiv), Cp_2_ZrHCl (2.0 equiv), **2d** (1.0 equiv), CuL* as specified (0.1 equiv), additive as specified (1.0 equiv or 5.0 equiv), in specified solvent (2.0 mL), room temperature. ee determined by HPLC. For more information on the procedures see Supporting Information File 1. ● (wave 1, entries 1–17), ♦ (wave 2, entries 18–30), ▼ (wave 3, entries 31–38).

Nevertheless, a Principal Component Analysis using JMP^®^ 12.1.0 (SAS) in 3 waves was carried out using **2d** as the starting material. In each experiment, the most promising variables were chosen based on results from previously published methods, the procedure optimised for **2a** (above), and the results of previous waves. The first wave was as a third factorial design with 3 categories: Ligand (**A**, **B**, **C**, **F** and **G**), counter-ion (ClO_4_^−^, I^−^ and OTf^−^) and solvent (CH_2_Cl_2_, Et_2_O and TBME, Table 1, entries 1–17; ● Figure 1, for more details see Supplorting Information File 1).

This first DoE study suggested that neither CuI nor TBME were good fits for the reaction (both consistently giving low ee). The combination of CuOTf in CH_2_Cl_2_ gave the best enantioselectivity (up to 83% ee, Table S1 entry 3, Supporting Information File 1) with ligand **G**. Unlike with **2a**, CuClO_4_ did not give high ee with **2d**; the highest value obtained was 43% ee (Table S1, entry 14). Interestingly Et_2_O gave mixed results with some low (e.g., 1% ee, Table S1, entry 1) and moderate (e.g., 56% ee, Table S1, entry 6) ee values obtained.

Based on those results, a second wave of DoE was designed as a 6^th^ factorial design with 4 factors: Ligand (**G**, **H**, **I**, **J**), counter-ion (OTf and NTf_2_), solvent (CH_2_Cl_2_, Et_2_O and CHCl_3_) and TMSCl equivalent (0, 1 and 5) (Table S1, entries 18–30, ♦). As mixed results were obtained with Et_2_O, we decided to investigate it more thoroughly. This second study emphasizes the intrinsic challenge of finding optimum conditions in complex asymmetric reactions. Whereas CuOTf seems to work best with CH_2_Cl_2_ as a solvent, CuNTf_2_ gave better enantioselectivity in Et_2_O. CHCl_3_ consistently provided lower enantioselectivity than CH_2_Cl_2_. In the small selection of ligands examined, **G** generally gave better results.

We designed a final study to investigate the role of various equivalents of additive (TMSCl and B(OiPr)_3_) with CuOTf and CuNTf_2_ in their respective favoured solvents (CH_2_Cl_2_ and Et_2_O) (Table S1, entries 31–38, ▼). B(OiPr)_3_ significantly lowered the ee (44% ee, Table S1, entry 33). The influence of TMSCl on the reaction was highly dependent on the other reaction parameters; CuNTf_2_ in Et_2_O with no additive gave 67% ee (Table S1, entry 34), while adding 1 equiv of TMSCl gave a slight improvement (73% ee, Table S1, entry 35) but no further improvement was observed by adding more TMSCl (5 equiv, 74% ee, Table S1, entry 36). On the other hand, using 1 equiv of TMSCl with CuOTf in CH_2_Cl_2_ did not modify the ee (81% ee, Table S1, entry 32), while adding 5 equiv of TMSCl was detrimental to enantioselectivity (60% ee, Table S1, entry 31).

Despite our efforts to optimise this second system, the highest enantioselectivity obtained was 83% ee, which is the same as for allyl chloride **2a**. It became clear that when using alkylzirconocene nucleophiles and Cu catalysis, derivatised 3,6-dihydro-2*H*-pyrans are difficult to obtain in high enantiomeric excess. Moreover, both optimised systems gave poor yield; 25% yield with 100% conversion from allyl chloride **2a** and 17% yield with 31% conversion from allyl phosphate **2d**.

**Scheme 2:**
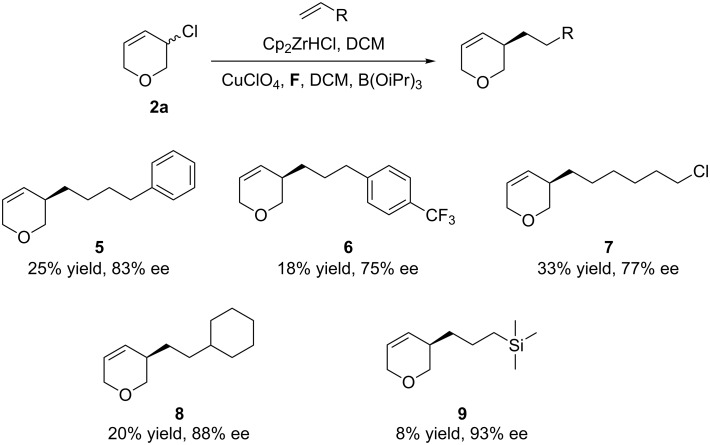
Scope of nucleophiles. Conditions: alkene (2.5 equiv), Cp_2_ZrHCl (2.0 equiv), 3-chloro-3,6-dihydro-2*H*-pyran **2a** (1.0 equiv), CuCl (10 mol %), **D** (10 mol %), AgClO_4_ (10 mol %), B(OiPr)_3_ (1.0 equiv), in CH_2_Cl_2_ (2.0 mL), room temperature. Isolated yield. ee determined by HPLC or GC. For more information see Supporting Information File 1.

Various alkenes were examined using the allyl chloride **2a** system (Scheme 2). The reaction showed tolerance in functional groups such as CF_3_ (**6**, 75% ee) Cl (**7**, 77% ee), and cyclohexane (**8**, 88% ee). Electron rich allyl silane could also be used to introduce a TMS group (**9**, 93% ee), but all the yields were poor.

**Figure 2:**
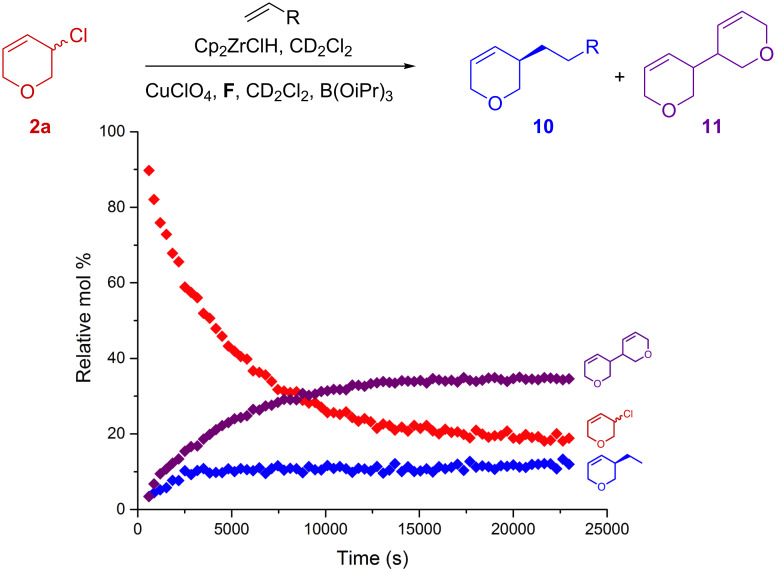
Reaction kinetics as monitored by in situ ^1^H NMR spectroscopy from 3-chloro-3,6-dihydro-2*H*-pyran (**2a**).

**Figure 3:**
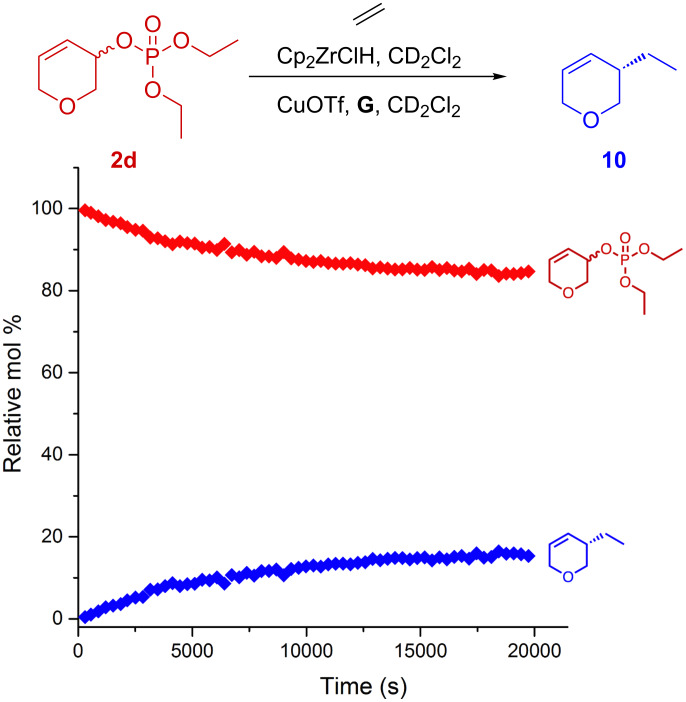
Reaction kinetics as monitored by in situ ^1^H NMR spectroscopy from 3,6-dihydro-2*H*-pyran-3-yl diethyl phosphate (**2d**).

To investigate why we obtained such poor yields, and possibly shed light onto the reaction mechanism, we decided to follow both reactions in time using in situ NMR spectroscopy (Figure 2 and Figure 3). Reactions were carried out as normal, but in deuterated solvents and mixed in an NMR tube (see Supporting Information File 1). Ethylene was used as the alkylzirconium precursor as it greatly simplifies the NMR spectra. Spectra were recorded at regular intervals over time where relative concentrations are based on integration of the best resolved ^1^H signal for each species and calibrated accordingly.

Through these kinetic studies, it is clear that the allyl chloride **2a** system fails because the starting material dimerises to give **11** as the major reaction product (60% isolated yield – 30 mol % by NMR) (Figure 2). This is consistent with the observed ≈100% conversion but low product yields. Presumably **11** arises from the homocoupling of allyl chloride **2a**, possibly through a π-allyl-Cu intermediate [39–43]. Although both the conditions and leaving groups differ in the two reactions it is not clear why **2a**, but not **2d**, dimerizes. **11** can exists as 3 isomers, a meso compound and two enantiomers. Upon comparison to literature data [44], we concluded that we form a mixture of all three, as a 1:1 mixture of the meso and racemic material. Our samples did not rotate plane polarized light, emphasizing the racemic nature of the sample and suggesting that **11** is formed in a completely non-selective pathway.

In the case of allyl phosphate **2d**, the system appears to lack reactivity and the reaction quickly dies, so that **10** (Figure 3) is formed with poor conversion, and we speculate that the phosphate leaving group inhibits the catalyst which would explain why only ~10% of product is formed.

**Figure 4:**
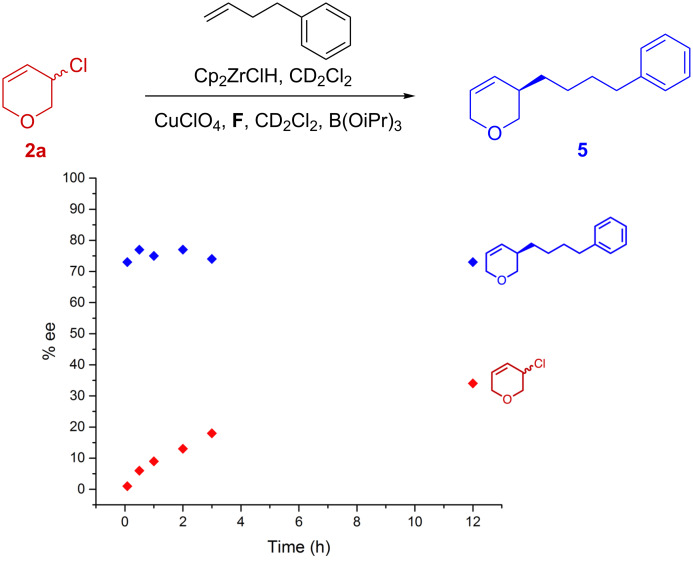
Kinetic ee analysis using **2a**. ee of reaction with 3-chloro-3,6-dihydro-2*H*-pyran (**2a**) as measured by removing aliquots in time.

**Figure 5:**
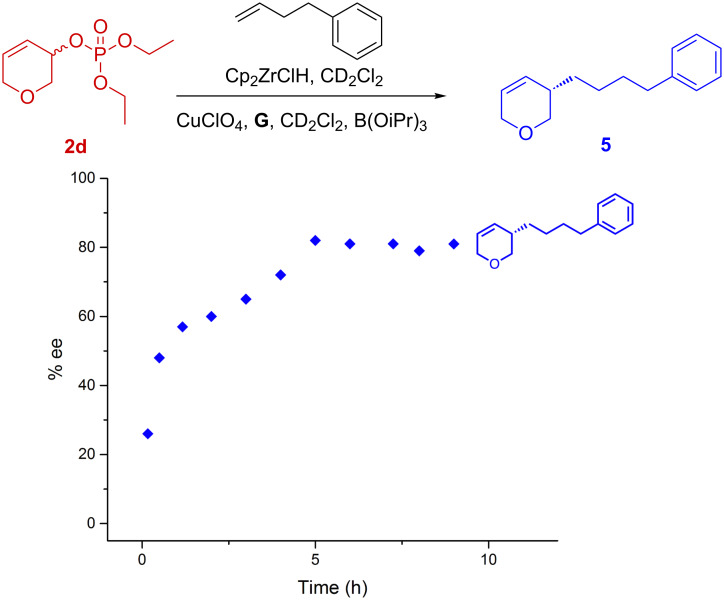
Kinetic ee analysis using **2d**. ee of reaction with 3,6-dihydro-2*H*-pyran-3-yl diethyl phosphate (**2d**) as measured by removing aliquots in time.

To obtain further mechanistic information we followed the ee of these reactions in time (Figure 4 and Figure 5). In the system using chloride **2a**, the ee of product **5** remains constant throughout the reaction (~75% ee, Figure 4). Starting chloride **2a** was found to be quite robust so that we could also determine its enantiomeric excess during the course of the reaction. Initially **2a** is racemic but it becomes scalemic to slowly reach 34% ee when the reaction is complete (~12 hours). From these observations and our experimental demonstration that **2a** is much more stable than all-carbocyclic **1**, it appears that **2a** undergoes kinetic resolution. However, this system is clearly complicated by the fact that the majority of **2a** is consumed during byproduct **11**'s formation.

In the phosphate system based on **2d**, the ee of product **5** was found to increase during the course of the reaction (Figure 5) so that **5** was ~26% ee after a few minutes, and increased to ~82% ee after 5 hours. Unfortunately, analytical conditions to separate the enantiomers of **2d**, so we could measure the enantiomeric excess of this starting material, could not be found. At this stage it is not possible to provide a full mechanistic rationalization of these reactions. It is also not immediately obvious how to improve yields and enantiomeric excesses. The kinetic studies suggest that the two reactions work through very different mechanisms and it strikes us as remarkable how both systems give roughly the same enantioselectivity and poor yield, yet have significantly different pathways.

## Conclusion

The Cu-catalyzed AAA of alkylzirconium reagents to racemic heterocyclic electrophiles was explored. After extensive examination, two different methods for obtaining 3,6-dihydro-2*H*-pyran derivatives with respectable levels of ee (≈83% ee) were developed. Unfortunately, the yields were poor in both cases. Kinetic studies were performed to help to understand the difficulties associated with these reactions. While we were not able to resolve the issues of yield in these studies, this work reveals remarkable mechanistic diversity in Cu-catalysed asymmetric alkylation reactions to racemic starting materials.

## Supporting Information

File 1 Additional material.
