# Supplementary material for: Copper-catalysed asymmetric allylic alkylation of alkylzirconocenes to racemic 3,6-dihydro-2H-pyrans
Source: Beilstein J Org Chem. 2015 Dec 3;11:2435–43. doi: 10.3762/bjoc.11.264 (PMC4685918; doi:10.3762/bjoc.11.264)
Supplement: File 1 — Additional material. [file Beilstein_J_Org_Chem-11-2435-s001.pdf]

Supporting Information

for

Copper-catalysed asymmetric allylic alkylation of alkylzirconocenes to racemic 3,6-dihydro-2*H*-pyrans

Emeline Rideau and Stephen P. Fletcher*

Address: Department of Chemistry, Chemistry Research Laboratory, University of Oxford, 12 Mansfield Road, Oxford, OX1 3TA, UK, Fax: +44(1865)285002

Email: Stephen P. Fletcher - stephen.fletcher@chem.ox.ac.uk

*Corresponding author

Additional Material

Table of Contents

**I.**	**General Information**	**S3**
**II.**	**Chemicals**	**S3**
**III.**	**Synthesis of starting materials**	**S4**
	1-(Allyloxy)pent-4-en-2-ol	S4
	3,6-Dihydro-2*H*-pyran-3-ol	S5
	3-Chloro-3,6-dihydro-2*H*-pyran (**2a**)	S6
	3-Bromo-3,6-dihydro-2*H*-pyran (**2b**)	S7
	3,6-Dihydro-2*H*-pyran-3-yl acetate (**2c**)	S9
	3,6-Dihydro-2*H*-pyran-3-yl diethyl phosphate (**2d**)	S10
**IV.**	**General Procedures**	**S1**
	Procedures of enantioenriched products from **2a**	S11
	Procedures of enantioenriched products from **2d**	S12
	Procedures of racemic products from **2a**	S12
	Procedures of racemic products from **2d**	S13
	Derivatisation of products to the corresponding epoxides for GC analysis	S13
**V.**	**Design of experiments using 2d**	**S13**
**VI.**	**Specific Procedures**	**S15**
	3-(4-Phenylbutyl)-3,6-dihydro-2*H*-pyran (**5**)	S15
	3-(3-(4-(Trifluoromethyl)phenyl)propyl)-3,6-dihydro-2*H*-pyran (**6**)	S18
	3-(6-Chlorohexyl)-3,6-dihydro-2*H*-pyran (**7**)	S20
	3-(2-Cyclohexylethyl)-3,6-dihydro-2*H*-pyran (**8**)	S22
	(3-(3,6-Dihydro-2*H*-pyran-3-yl)propyl)trimethylsilane (**9**)	S24
**VII.**	**Side-product**	**S26**
	3,3',6,6'-Tetrahydro-2*H*,2'*H*-3,3'-bipyran (**10**)	S27
	Racemic synthesis of 3,3',6,6'-tetrahydro-2*H*,2'*H*-3,3'-bipyran (**10**)	S28
**VIII.**	**Mechanistic experiments**	**S28**
	3-Chloro-3,6-dihydro-2*H*-pyran (**2a**)	S29
	Kinetic NMR	S29
	Kinetic ee	S30
	3,6-Dihydro-2*H*-pyran-3-yl diethyl phosphate (**2d**)	S35
	Kinetic NMR	S35
	Kinetic ee	S36
**IX.**	**References**	**S39**

I. General Information

All reactions involving oxygen/moisture sensitive reagents were performed with anhydrous solvents in flame-dried glassware under a positive pressure of anhydrous argon, using standard Schlenk techniques. Cooling of reaction mixtures to −78 ºC was effected using an acetone/dry ice bath; to 0 °C using an ice/water bath; to other temperatures using a Julabo FT902 immersion cooler. Heating was performed using Drysyn® heating blocks.

In the cases where silver salts were used, the resulting solutions were filtered using syringe filters PTFE (0.2 μm, 13 mm diameter) from Camlab.

Analytical thin-layer chromatography was performed on glassplates pre-coated with silica gel (Silica Gel 60 F_{254}; Merck). Plates were visualised using UV light (λ = 254 nm) and then stained with either aqueous ceric ammonium molybdate (CAM), aqueous basic potassium permanganate ($KMnO_4$) or anysaldehyde and developed upon heating.

Flash chromatography was performed using silica gel (Apollo Scientific 60 (40-63 μm), Sigma Aldrich (Davisil®grade 636, pore size 60 Å, 35–60 mesh), Merck 60 Å or VWR (40–63 μm)). Pressure was applied at the column head via a flow of nitrogen with the solvent system used in parentheses.

Nuclear magnetic resonance spectra were acquired in deuterated solvents at room temperature on Bruker: AVIIIHD 400 nanobay, AVIIIHD 500, AVII 500, AVII 500 with cryoprobe spectrometers. Chemical shifts (δ) are reported in ppm from the residual solvent. Coupling constants (*J*) are quoted in Hertz (Hz) and are recorded to the nearest 0.1 Hz. Resonances are descried as singlet (s), doublet (d), triplet (t), quartet (q), quintet (quint), doublet of doublets (dd), doublet of doublets of doublets (ddd), doublet of triplets (dt), multiplet (m) and broad (br.). Labels Hb and Ha refer to diastereotopic protons attached to the same carbon and impart no stereochemical information. Assignments were made with the assistance of gCOSY, DEPT-Q, gHSQC and gHMBC NMR spectra.

Low resolution (LRMS) and high resolution (HRMS) mass spectral analyses were acquired by electrospray ionisation (ESI), electron impact (EI), Field Ionisation (FI). Low resolution ESI were recorded using an Agilent 6120 quadrupol LC/MS. High resolution accurate ESI were recorded using a Thermo Exactive 1.1 SP5 Benchtop orbitrap MS and EI/FI on a Waters GTC Temperature programmed solid probe inlet within the department of chemistry, University of Oxford.

Infrared spectroscopy (IR) measurements (neat, thin film) were carried out using a Bruker Tensor 27 FT-IR with internal calibration in the range of 4000–600 cm^{-1}. Absorption maxima are reported as wavenumbers (cm^{-1}).

Optical rotations were recorded using a Schmidt Haensch Unipol L 2000 Polarimeter.

Chiral HPLC separations were achieved using an Agilent 1260 Infinity series normal phase HPLC unit and HP Chemstation software. Chiralpak® columns (250 × 4.6 mm), fitted with matching Chiralpak® Guard Cartridges (10 × 4 mm), were used as specified. Solvents used were of HPLC grade (Fisher Scientific, Sigma Alrich or Rathburn). All eluent systems were isocratic.

II. Chemicals

WARNING: Perchlorates are explosive and should be handled with caution.

Chemicals and reagents were obtained from Sigma Aldrich, Alfa-Aesar, Apollo Scientific, Strem chemicals, Acros Organics, TCI UK and fluorochem, and were used as received unless otherwise stated. Deuterated solvents were purchased from Sigma-Aldrich ($CDCl_3$) and Fluorochem (CD_2Cl_2).

Dry dichloromethane (DCM), diethyl ether (Et_2O), tetrahydrofuran (THF), benzene and acetonitrile were collected fresh from a solvent purification system (MBraun, SPS-800) having been passed through anhydrous alumina columns. Chloroform ($CHCl_3$) was obtained by filtration through activated alumina (powder ~150 mesh, pore size 58 Å, basic, Sigma-Aldrich) columns. Dry *tert*-butyl methyl ether, 2-Me THF, acetone and MeOH were purchased from Acros Organics with an AcroSeal® or Sigma Aldrich with a similar sela as advertised dry solvent stored under inert atmosphere.

TMSCl was distilled before use and stored in Schlenk flasks under an argon atmosphere containing $CaCl_2$. Schwartz reagent was prepared according to a literature procedure[1] from Cp_2ZrCl_2 provided by Strem Chemicals. $(CuOTf)_2$.PhH was synthesised using a modified literature[2] procedure and carefully maintained under inter atmosphere. $(CuOTf)_2$.PhH should be a white or off-white powder. Phosphoramidite ligands were synthesised according to in-house procedures.[3]

III. Synthesis of starting materials

1-(Allyloxy)pent-4-en-2-ol

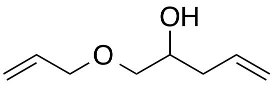

In analogy to a published procedure[4], prop-2-en-1-ol (3.4 mL, 50 mmol, 2.0 equiv) was added to a solution of butadiene monoxide (2.0 mL, 25 mmol, 1.0 equiv) in DMF (50 mL). Sodium hydride (2.00 g, 60% w/w in mineral oil, 50 mmol, 2.0 equiv) was added in 1 g portions at 0 °C The resulting mixture was stirred for an additional 30 min at 0 °C, and then overnight at 50 °C. The reaction mixture was quench at 0 °C with HCl (aq) (100 mL, 1 M) and stirred for 1 h to cool down. The resulting orange aqueous layer was extracted with ether (3 × 50 mL). The combined organic layers were washed with LiCl (10% w/w, 100 mL), brine (100 mL), dried over $MgSO_4$, filtered and concentrated under reduced pressure to afford the crude product as a yellow oil. Purification by flash column chromatography (SiO_2, Et_2O:pentane 30:70) yielded the title product as a yellow oil (2.1 g, 16.4 mmol, 66%).

^{1}H NMR (400 MHz, $CDCl_3$) δ_H /ppm 5.97-5.78 (m, 2H), 5.36 (d, 17.0 Hz, 1H), 5.28 (d, 17.0 Hz, 1H), 5.20 (d, 10.9 Hz, 2H), 4.32 (br. s, 1H), 4.04 (d, 5.8 Hz, 2H), 3.51 (dd, 9.6, 3.3 Hz, 1H), 3.33 (dd, 9.8, 8.2 Hz, 1H), 2.44 (br s, 1H).

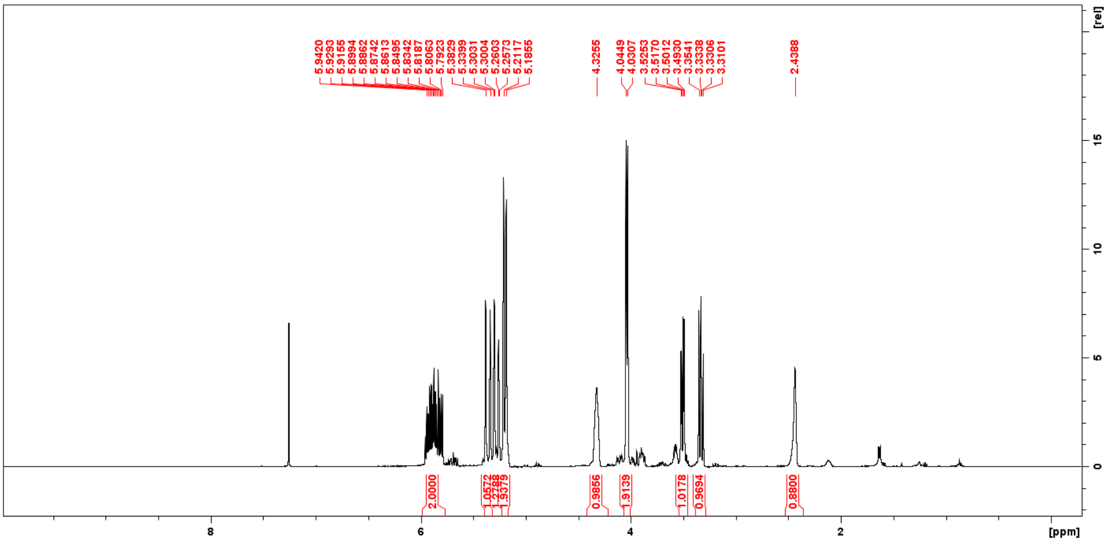

^{13}C NMR (100 MHz, $CDCl_3$) δ_C /ppm 136.7, 134.5, 117.6, 116.6, 74.0, 72.4, 71.6.

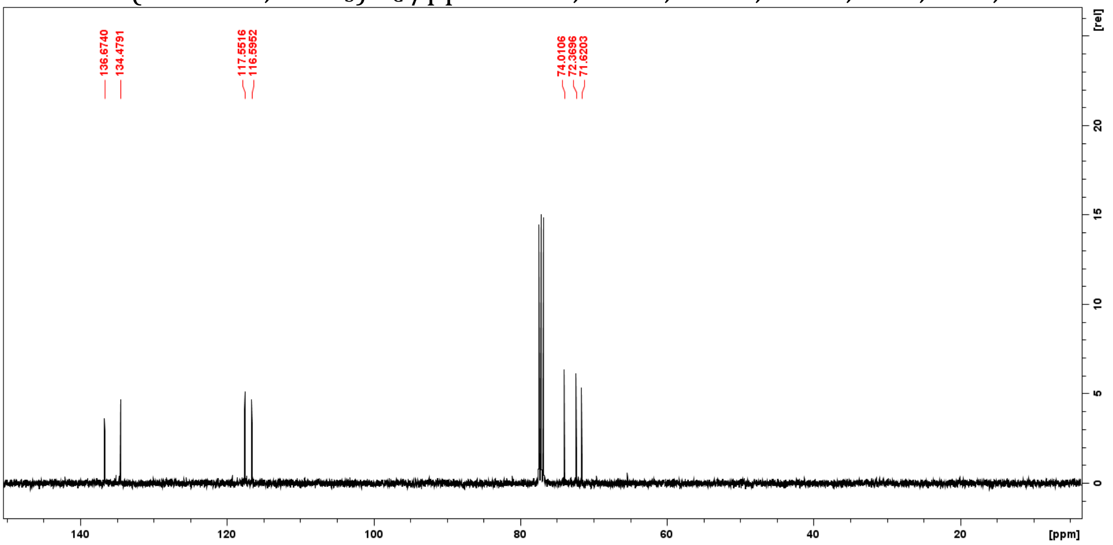

HRMS (ESI) *m*/z calcd for $C_7H_{12}O_2Na$ $[M+Na]^+$: 151.07295, found: 151.07286.

IR (ATR) (ν_{max}/cm^{-1}) 3422, 2924, 2857, 2360, 1424, 1104, 992, 927.

3,6-Dihydro-2*H*-pyran-3-ol

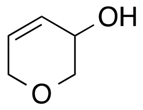

In analogy to a published procedure[4], Grubbs catalyst 1st generation (310 mg, 0.37 mmol, 0.02 equiv) was added to a solution of 1-(allyloxy)pent-4-en-2-ol (2.0 g, 15.6 mmol, 1.0 equiv) in DCM (150 mL) at room temperature and stirred overnight. The resulting reaction mixture was concentrated under reduced pressure to obtain the crude product as a brown oil. Purification by

flash column chromatography (SiO_2, Et_2O 50→100% in pentane) yielded the title product as a brown oil (1.4 g, 14.3 mmol, 92%).

^{1}H NMR (400 MHz, $CDCl_3$) δ_H /ppm 5.98-5.83 (m, 2H), 4.11 (d, 16.6 Hz, 1H), 4.00 (dd, 16.6 Hz, 2.0 Hz, 1H), 3.91 (dd, 6.1 Hz, 3.2 Hz, 1H), 3.79 (dd, 11.7 Hz, 2.6 Hz, 1H), 3.68 (dd, 11.7 Hz, 3.2 Hz, 1H), 1.86-1.79 (m, 1H).

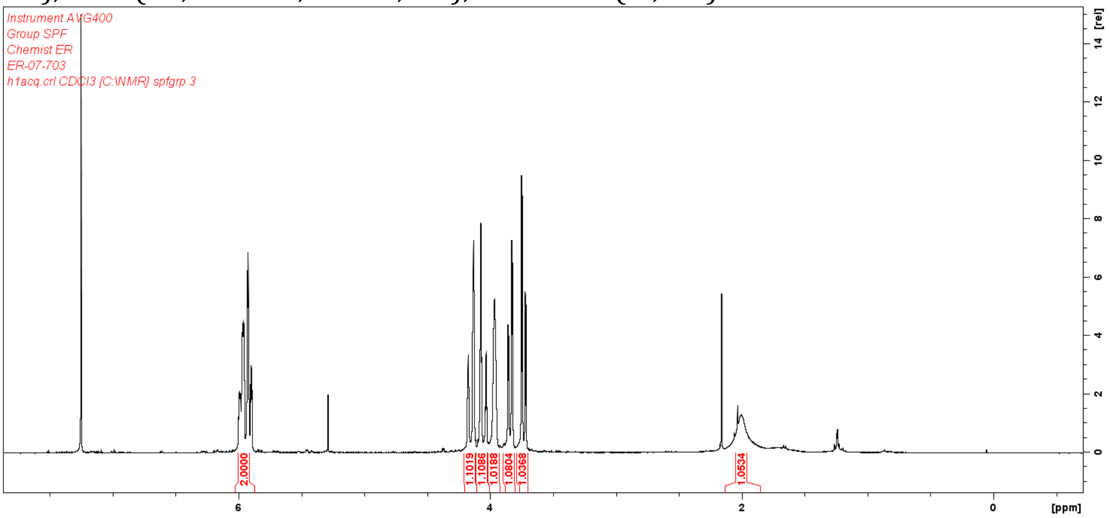

^{13}C NMR (100 MHz, $CDCl_3$) δ_C /ppm 130.0, 126.7, 70.8, 65.4, 62.7.

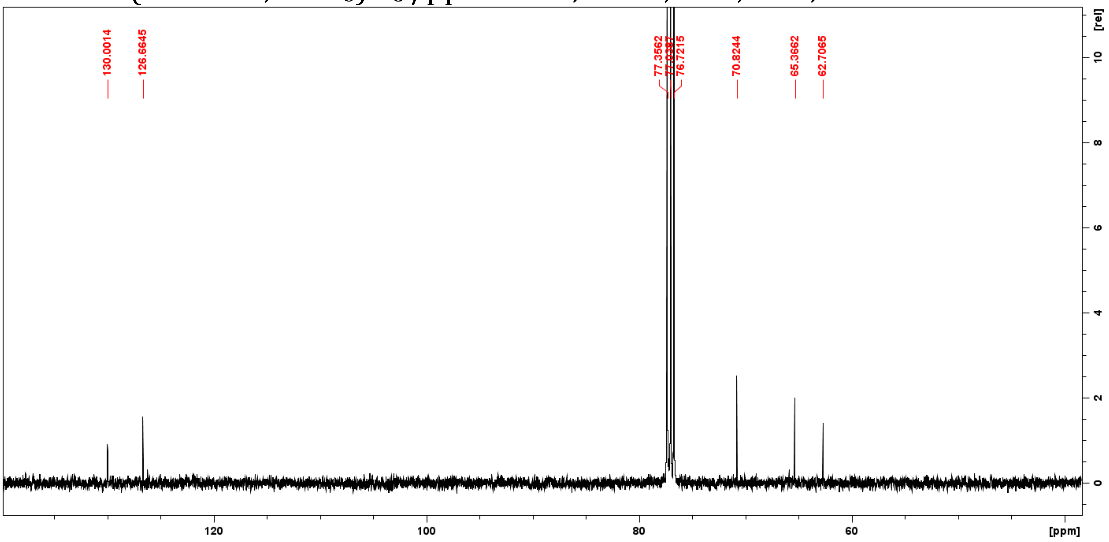

HRMS (ESI) *m*/z calcd for $C_5H_8O_2Na$ $[M+Na]^+$: 123.04165, found: 123.04143.

IR (ATR) (ν_{max}/cm^{-1}) 3381, 2846, 1226, 1086, 1064, 1014, 937, 835, 817, 695.

3-Chloro-3,6-dihydro-2*H*-pyran (2a)

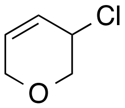

Based on a modified procedure [4], PCl_3 (1.6 mL, 18.5 mmol, 0.37 equiv) was added dropwise to a mixture of 3,6-dihydro-2*H*-pyran-3-ol (5.0 g, 49.9 mmol, 1 equiv) and pyridine (0.4 mL, 5.0 mmol, 0.1 equiv) at 0 °C. After stirring for 30 min at 0 °C, the reaction mixture was allowed to warm up to room temperature and stirred overnight. The non-viscous organic layer of the resulting mixture was removed by pipette and the bottom layer was rinsed with pentane (3 × 1 mL). The combined organic material was concentrated under reduced pressure and purified by Kugelrohr distillation (20 mbar, 100 °C) to afford the title product as a very pale yellow liquid (4.1 g, 34.6 mmol, 69%)

^{1}H NMR (400 MHz, $CDCl_3$) δ_H /ppm 5.97-5.88 (m, 2H), 4.52-4.45 (m, 1H), 4.23 (ddd, *J*= 16.8, 3.8, 2.2 Hz, 1H), 4.12 (ddd, *J*= 16.8, 3.8, 1.9 Hz, 1H), 4.04 (dd, *J*= 12.1, 4.0 Hz, 1H), 3.84 (dd, *J*= 12.1, 5.0 Hz, 1H).

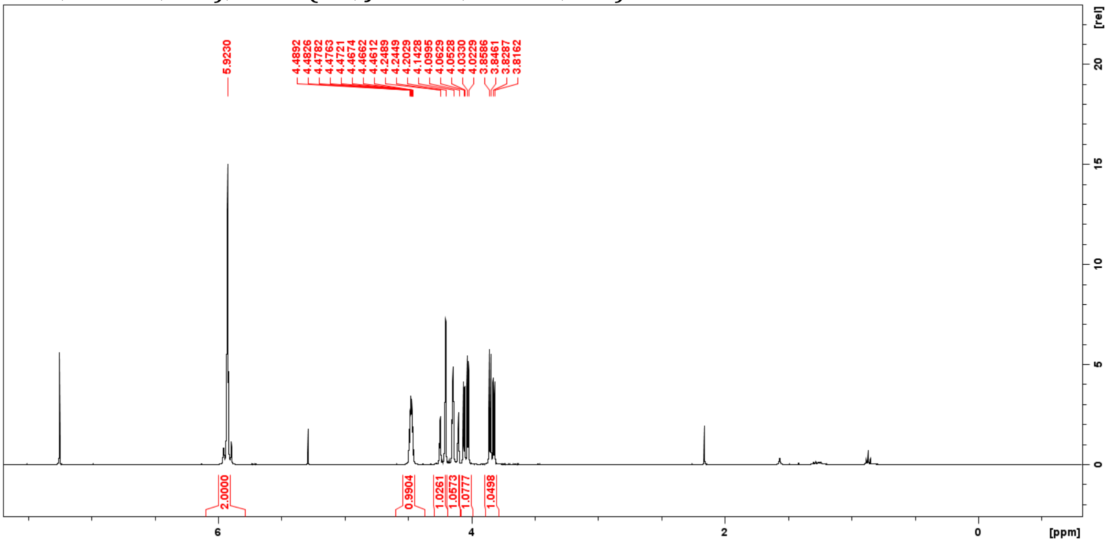

^{13}C NMR (100 MHz, $CDCl_3$) δ_C /ppm 129.8, 126.0, 70.1, 65.1, 51.0.

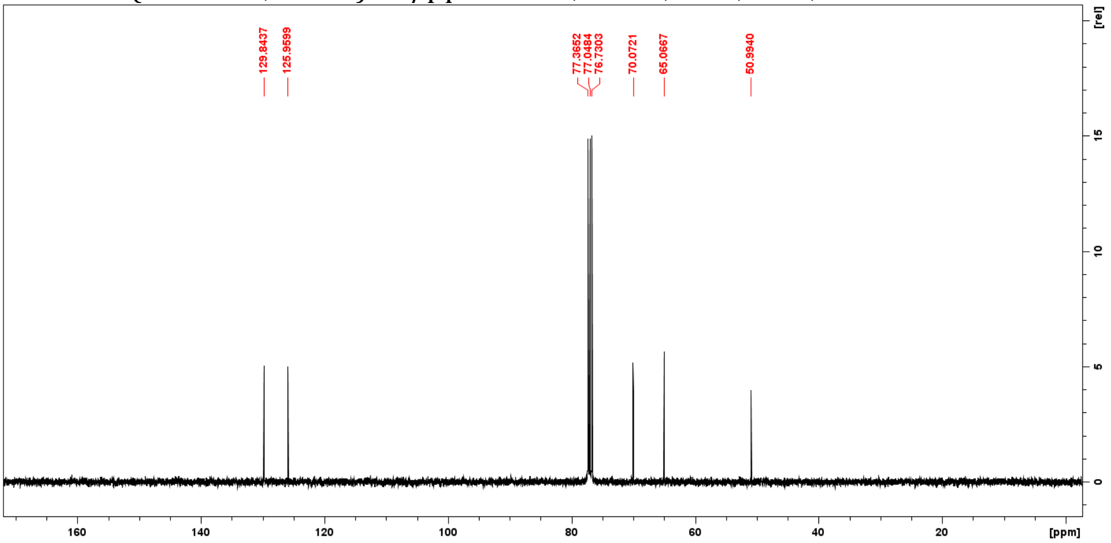

HRMS (EI/FI) *m*/z calcd for C_5H_7OCl $[M]^+$:118.0185, found:118.0185.

IR (ATR) (ν_{max}/cm^{-1}) 2833, 1289, 1114, 1094, 997, 829, 735.

Spectral data was in accordance with literature[4].

3-Bromo-3,6-dihydro-2*H*-pyran (2b)

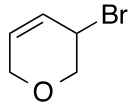

In analogy to the 3-chloro-3,6-dihydro-2*H*-pyran procedure, PBr_3 (180 µL, 1.89 mmol, 0.37 equiv) was added dropwise to a mixture of 3,6-dihydro-2*H*-pyran-3-ol (0.5 g, 4.99 mmol, 0.98 equiv) and pyridine (41 µL, 0.50 mmol, 0.1 equiv) at 0 °C. After stirring for 30 min at 0 °C, the reaction mixture was allowed to warm to room temperature and stirred overnight. The non-viscous organic layer of the resulting mixture was removed by pipette and the bottom layer was rinsed with pentane (3 × 1 mL). The combined organic materials was concentrated under reduced pressure and purified by Kugelrohr distillation (57 mbar, 140 °C) to afford the title product as an orange oil (296 mg, 1.81 mmol, 36%)

^{1}H NMR (400 MHz, $CDCl_3$) δ_H /ppm6.07-6.00 (m, 1H), 5.90-5.83 (m, 1H), 4.63 (br s, 1H), 4.31 (ddd, 17.3, 4.4, 2.2 Hz, 1H), 4.20 (ddd, 17.3, 4.6, 2.4 Hz, 1H), 4.07 (dd, 12.2, 3.9, 1H), 3.98 (dd, 12.2, 4.6, 1H).

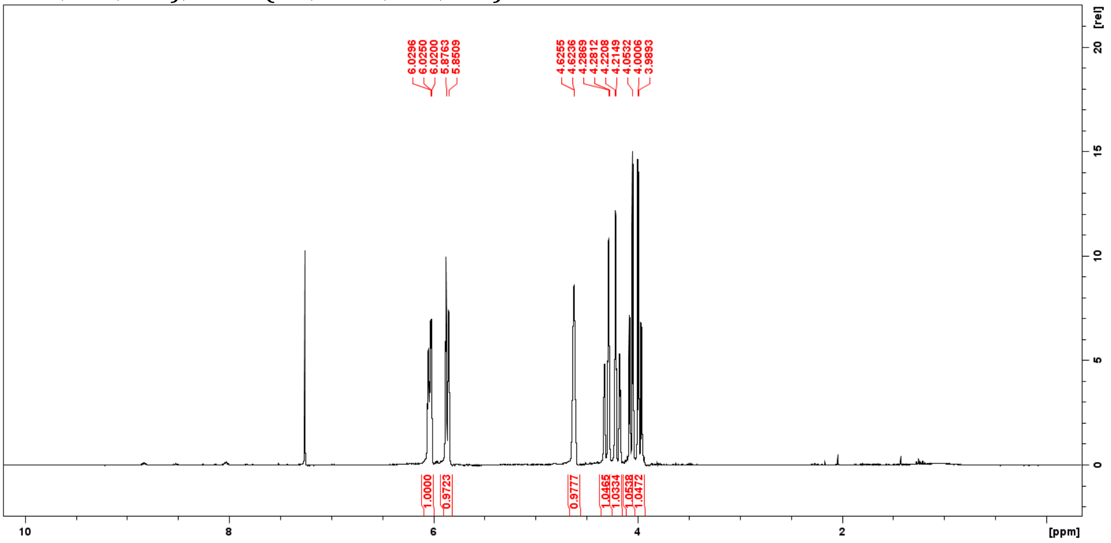

^{13}**C NMR** (100 MHz, $CDCl_3$) δ_C /ppm 129.6, 126.7, 70.3, 65.1, 43.0.

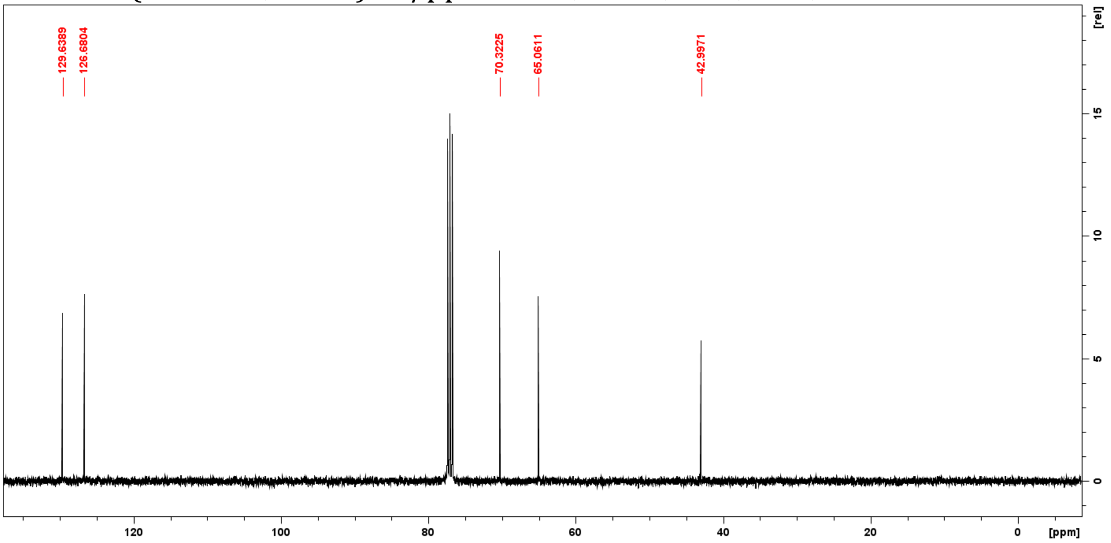

HRMS (EI/FI) *m*/z calcd for C_5H_7OBr $[M]^+$: 161.9680, found: 161.9685.

IR (ATR) (ν_{max}/cm^{-1}) 2867, 1307, 1156, 987, 795, 624.

3,6-dihydro-2*H*-pyran-3-yl acetate (2c)

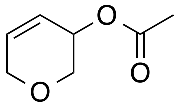

According to a modified procedure[5], 6-dihydro-2*H*-pyran-3-ol (0.20 g, 2.0 mmol, 1.0 equiv) was dissolved in Et_2O (2.0 mL). Pyridine (0.2 mL, 3.0 mmol, 1.5 equiv) DMAP (243 mg, 2.0 mmol, 1.0 equiv) and then acetic anhydride (380 μL, 4.0 mmol, 2.0 equiv) were added dropwise to the stirring yellow solution. The reaction mixture was stirred overnight. The reaction was diluted in Et_2O (10 mL) and quenched with H_2O (2 × 15 mL). The mixture was partitioned and the aqueous phase was extracted with Et_2O (3 × 10 mL). The combined organic extracts were washed with brine (20 mL), dried over $MgSO_4$, filtered and concentrated under reduced pressure. Purification by flash column chromatography (SiO_2, Et_2O 20% in petrol) yielded the title product as a yellow oil (106 mg, 0.746 mmol, 37%)

^{1}**H NMR** (400 MHz, $CDCl_3$) δ_H /ppm 6.10-6.04 (m, 1H), 5.96-5.89 (m, 1H), 5.11-5.06 (m, 1H), 4.22 (d, 16.9 Hz, 1H), 4.08 (ddd, 16.9, 5.4, 2.2 Hz, 1H), 3.92 (dd, 12.5, 2.5 Hz, 1H), 3.80 (dd, 12.5, 3.4 Hz, 1H), 2.09 (s, 3H).

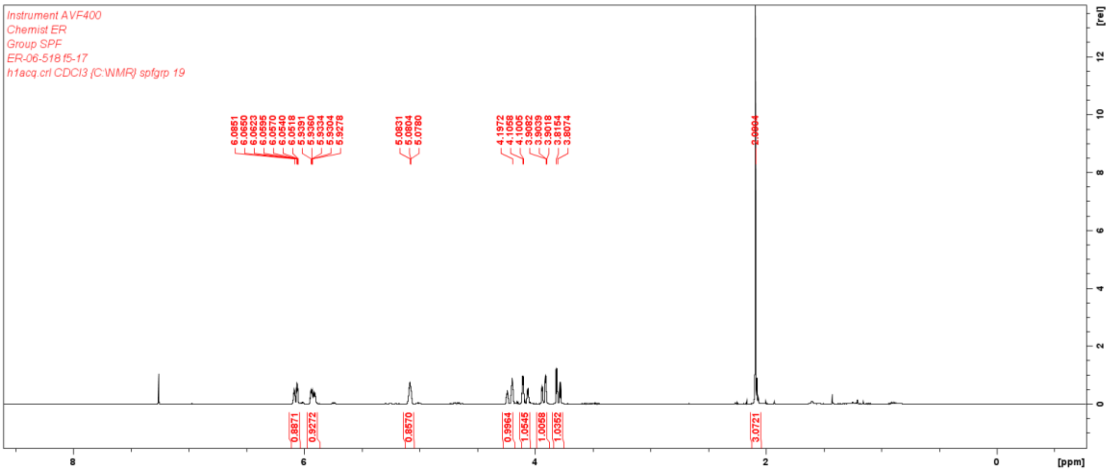

^{13}**C NMR** (100 MHz, $CDCl_3$) δ_C /ppm 170.8, 132.2, 122.5, 67.7, 65.0, 64.8, 21.2.

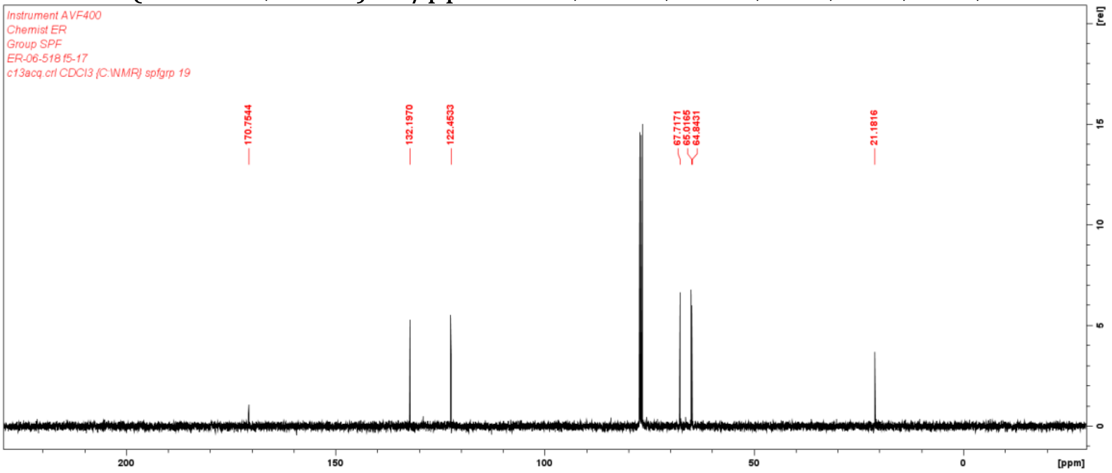

HRMS (ESI) *m*/z calcd for $C_7H_{10}O_3Na$ $[M+Na]^+$: 165.05222, found: 165.05219;

IR (ATR) (ν_{max}/cm^{-1}) 1727, 1371, 1227, 1188, 1097, 1063, 1020, 896, 846.

Spectral data is in accordance with literature[6].

3,6-Dihydro-2*H*-pyran-3-yl diethyl phosphate (2d)

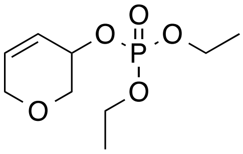

According to a modified procedure[7], diethyl chlorophosphate (2.4 mL, 16.5 mmol, 1.03 equiv) was added dropwise to a mixture of 6-dihydro-2*H*-pyran-3-ol (1.6 g, 16.0 mmol, 1.0 equiv) and pyridine (6.7 mL, 83.1 mmol, 5.2 equiv) in DCM (15 mL) at 0 °C. After stirring for 10 min at 0 °C, DMAP (0.39 g, 0.32 mmol, 0.02 equiv) was added to the reaction mixture. The reaction mixture was allowed to warm up to room temperature and stirred overnight. The reaction was diluted in DCM (10 mL) and quenched with H_2O (2 × 15 mL). The mixture was partitioned and the aqueous phase was extracted with DCM (3 × 10 mL). The combined organic extracts were washed with brine (20 mL), dried over $MgSO_4$,

filtered and concentrated under reduced pressure. Purification by flash column chromatography (SiO_2, Et_2O 100% and then 10% MeOH in $CHCl_3$) yielded the title product as a yellow oil (3.39 mg, 14.4 mmol, 90%)

^{1}H NMR (400 MHz, $CDCl_3$) δ_H /ppm 6.07-6.02 (m, 1H), 6.01-5.95 (m, 1H), 4.87-4.71 (m, 1H), 4.30-4.21 (m, 1H), 4.20-4.07 (m, 5H), 3.95 (dd, 12.5, 3.4 Hz, 1H), 3.85 (dd, 12.1, 3.6 Hz, 1H), 1.34 (tdd, 7.2, 1.3, 1.2 Hz, 6H).

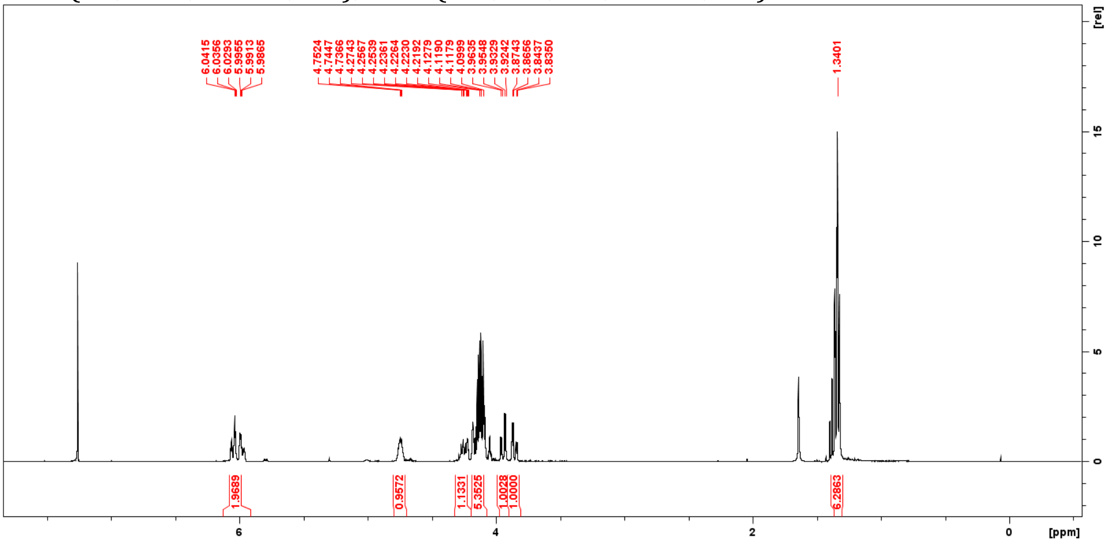

^{13}C NMR (100 MHz, $CDCl_3$) δ_C /ppm 131.9, 123.6 (d, *J*= 4.8 Hz), 68.5 (d, *J*= 5.6 Hz), 68.2 (d, *J*= 5.6 Hz), 65.1, 64.0 (d, *J*= 6.3 Hz), 16.2 (d, *J*= 7.3 Hz).

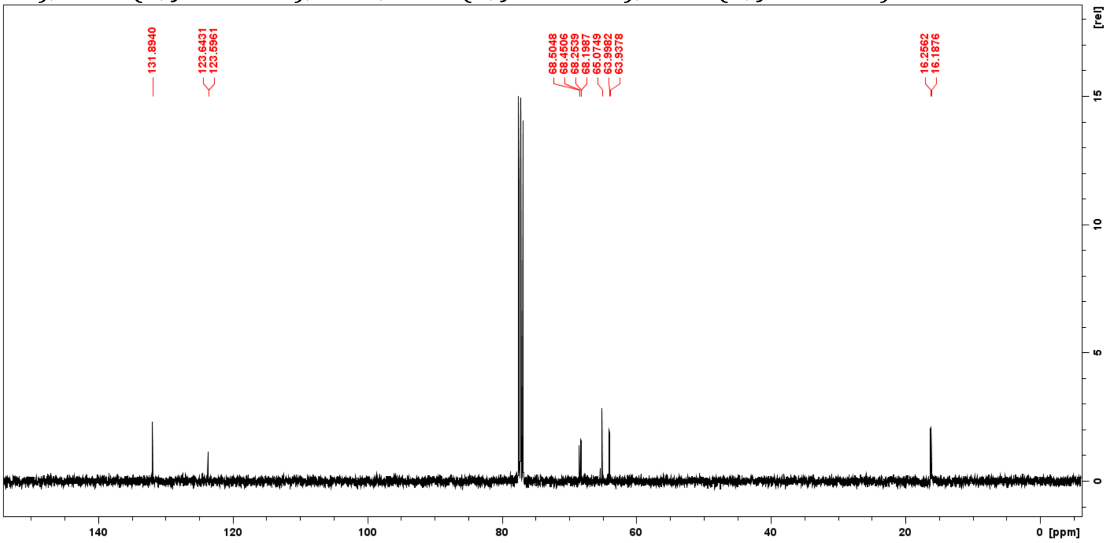

^{31}**P NMR** (200 MHz, $CDCl_3$) δ_C /ppm -0.01 (s, 1P).

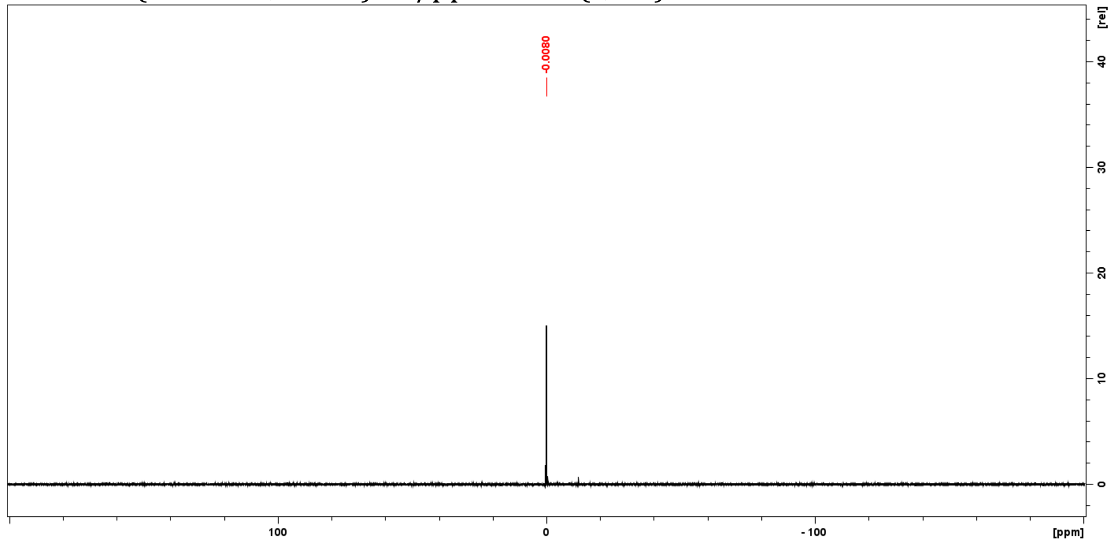

HRMS (ESI) *m*/z calcd for $C_9H_{18}O_5P$ $[M]^+$: 237.08864, found: 237.08840.

IR (ATR) (ν_{max}/cm^{-1}) 2984, 1262, 1006, 974, 800.

IV. General Procedures

Procedures of enantioenriched products from 2a

In a flame-dried flask under inert atmosphere, Cp_2ZrHCl (2.0 equiv) was added to a solution of alkene (2.5 equiv) in CH_2Cl_2 under an argon atmosphere and stirred vigorously until a clear yellow solution was obtained (20–40 min). Simultaneously, in another flask under inert atmosphere, CuCl (0.1 equiv) and (*R*)- **D** (0.1 equiv) were dissolved in CH_2Cl_2 and stirred for 1 h at room temperature. $AgClO_4$ (0.11 equiv ***Perchlorates are explosive and should be handled with caution***) was added to the freshly formed Cu-ligand complex solution and stirred for 15 min. The resulting catalyst complex mixture was filtered into the freshly prepared alkylzirconocene species. After 10 min, 3-chloro-3,6-dihydro-2*H*-pyran (1.0 equiv) was added dropwise via a microsyringe to the resulting black solution followed by the dropwise addition of $B(OiPr)_3$ (1.0 equiv). The reaction mixture was stirred overnight. The reaction mixture was diluted with Et_2O (2 mL) and quenched with NH_4Cl (3 mL, 1 M). The mixture was partitioned and the aqueous phase was extracted with Et_2O (3 × 10 mL). The combined organic extracts were washed with $NaHCO_3$ (aq., sat., 30 mL), dried over $MgSO_4$, filtered and concentrated under reduced pressure. Purification by flash column chromatography (SiO_2, Et_2O 0→2% in pentane) yielded the enantioenriched products.

Procedures of enantioenriched products from 2d

In a flame-dried flask under inert atmosphere, Cp_2ZrHCl (2.0 equiv) was added to a solution of alkene (2.5 equiv) in CH_2Cl_2 under an argon atmosphere and stirred vigorously until a clear yellow solution was obtained (20–40 min).

Simultaneously, in another flask under inert atmosphere, CuCl (0.1 equiv) and (*S, S*)- **A** (0.1 equiv) were dissolved in CH_2Cl_2 and stirred for 1 h at room temperature. AgOTf (0.11 equiv) was added to the freshly formed Cu-ligand complex solution and stirred for 15 min. The resulting catalyst complex mixture was filtered into the freshly prepared alkylzirconocene species. After 10 min, 3,6-dihydro-2*H*-pyran-3-yl diethyl phosphate (1.0 equiv) was added dropwise via a microsyringe to the resulting black solution and stirred overnight. The reaction mixture was diluted with Et_2O (2 mL) and quenched with NH_4Cl (3 mL, 1 M). The mixture was partitioned and the aqueous phase was extracted with Et_2O (3 × 10 mL). The combined organic extracts were washed with $NaHCO_3$ (aq., sat., 30 mL), dried over $MgSO_4$, filtered and concentrated under reduced pressure. Purification by flash column chromatography (SiO_2, Et_2O 0➔2% in pentane) yielded the enantioenriched products.

Procedures of racemic products from 2a

In a flame-dried flask under inert atmosphere, Cp_2ZrHCl (2.0 equiv) was added to a solution of alkene (2.5 equiv) in CH_2Cl_2 under an argon atmosphere and stirred vigorously until a clear yellow solution was obtained (20-40 min). Simultaneously, in another flask under inert atmosphere, CuCl (0.1 equiv) and (*S, S, S*)-Feringa ligand (0.05 equiv) and (*R, R, R*)-Feringa ligand (0.05 equiv) were dissolved in CH_2Cl_2 and stirred for 1 h at room temperature. $AgClO_4$ (0.11 equiv ***Perchlorates are explosive and should be handled with caution***) was added to the freshly formed Cu-ligand complex solution and stirred for 15 min. The resulting catalyst complex mixture was filtered into the freshly prepared alkylzirconocene species. After 10 min, 3-chloro-3,6-dihydro-2*H*-pyran (1.0 equiv) was added dropwise via a microsyringe to the resulting black solution followed by the dropwise addition of $B(OiPr)_3$ (1.0 equiv). The reaction mixture was stirred overnight. The reaction mixture was diluted with Et_2O (2 mL) and quenched with NH_4Cl (3 mL, 1 M). The mixture was partitioned and the aqueous phase was extracted with Et_2O (3 × 10 mL). The combined organic extracts were washed with $NaHCO_3$ (aq., sat., 30 mL), dried over $MgSO_4$, filtered and concentrated under reduced pressure. Purification by flash column chromatography (SiO_2, Et_2O 0➔2% in pentane) yielded the racemic products.

Procedures of racemic products from 2d

In a flame-dried flask under inert atmosphere, Cp_2ZrHCl (206.0 mg, 0.8 mmol, 2.0 equiv) was added to a solution of alkene (1.0 mmol, 2.5 equiv) in CH_2Cl_2 (0.40 mL) under an argon atmosphere and stirred vigorously until a clear yellow solution was obtained (20–40 min). The hydrozirconated mixture was dissolved in DCM (2.0 mL) and then CuBr.DMS (82 mg, 0.4 mmol, 1.0 equiv) was added. After 10 min, 3,6-dihydro-2*H*-pyran-3-yl diethyl phosphate (94.5 mg, 0.4 mmol, 1.0 equiv) was added dropwise via a microsyringe to the resulting black solution and stirred overnight. The reaction mixture was diluted with Et_2O (2 mL) and quenched with NH_4Cl (3 mL, 1 M). The mixture was partitioned and the aqueous phase was extracted with Et_2O (3 × 10 mL). The combined organic extracts were washed with $NaHCO_3$ (aq., sat., 30 mL), dried over $MgSO_4$, filtered

and concentrated under reduced pressure. Purification by flash column chromatography (SiO_2, Et_2O 0➔2% in pentane) yielded the racemic products.

Derivatisation of products to the corresponding epoxides for GC analysis

In a flame-dried flask, the isolated product (1.0 equiv) was dissolved in CH_2Cl_2 (6 mL, for 0.4 mmol scale reaction) under an argon atmosphere. *m*-CPBA (2.0 equiv) and Na_2HPO_4 (3.0 equiv) were added at room temperature and the reaction mixture was stirred arbitrarily for 3 h before being diluted and quenched by addition of Et_2O (10 mL) and an aqueous solution of saturated $Na_2S_2O_3$ (10 mL) . The organic layer was washed with NaOH (1 M aq., 3 × 5mL), dried over Mg_2SO_4, filtered and concentrated under vacuum. The crude mixture of diastereoisomeric epoxides was directly analyzed by GC chromatography using a chiral non-racemic stationary phase.

V. Design of experiments using 2d

Table S1. Design of experiments using 2d

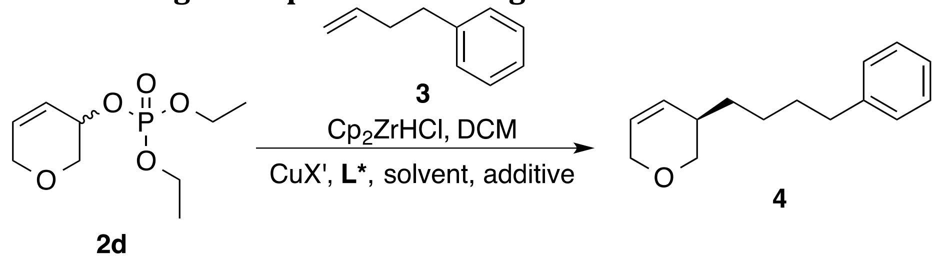

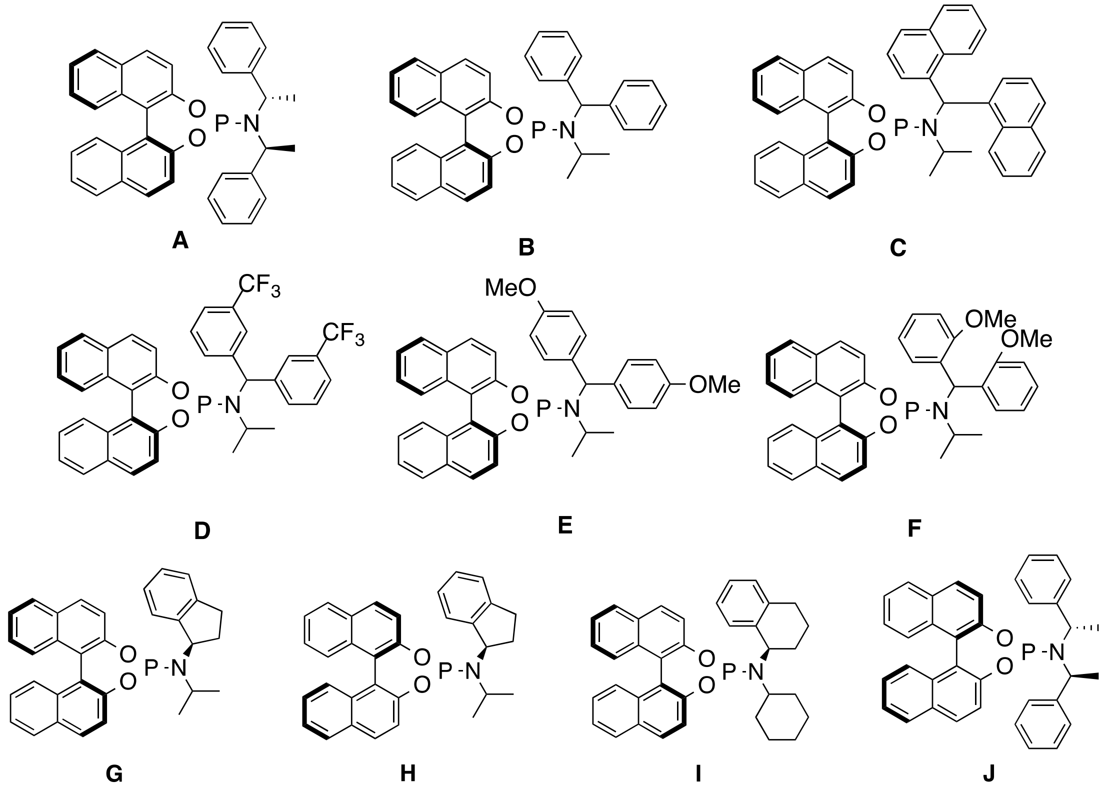

1st wave

Entry	Ligand	CuX	Solvent	ee
**1**	**A**	$CuClO_4$	Et_2O	**1%**
**2**	**A**	CuI	CH_2Cl_2	**29%**
**3**	**G**	CuOTf	CH_2Cl_2	**83%**
**4**	**G**	$CuClO_4$	TBME	**26%**
**5**	**B**	$CuClO_4$	CH_2Cl_2	**40%**
**6**	**C**	CuOTf	Et_2O	**56%**
**7**	**F**	CuI	Et_2O	**1%**
**8**	**F**	$CuClO_4$	TBME	**2%**
**9**	**B**	CuI	TBME	**7%**
**10**	**A**	CuOTf	TBME	**19%**
**11**	**F**	CuOTf	CH_2Cl_2	**74%**
**12**	**C**	CuOTf	CH_2Cl_2	**63%**
**13**	**G**	CuI	Et_2O	**13%**
**14**	**B**	$CuClO_4$	Et_2O	**43%**
**15**	**C**	CuI	TBME	**4%**
**16**	**C**	CuClO4	CH_2Cl_2	**43%**
**17**	**B**	CuOTf	Et_2O	**51%**

2nd wave

Entry	Ligand	CuX	Solvent	TMSCl (equiv)	ee
**18**	**I**	CuOTf	$CHCl_3$	5	**49%**
**19**	**G**	CuOTf	CH_2Cl_2	1	**83%**
**20**	**I**	$CuNTf_2$	Et_2O	1	**70%**
**21**	**H**	CuOTf	Et_2O	0	**29%**
**22**	**H**	$CuNTf_2$	$CHCl_3$	5	**41%**
**23**	**J**	$CuNTf_2$	CH_2Cl_2	5	**35%**
**24**	**G**	$CuNTf_2$	$CHCl_3$	0	**40%**
**25**	**I**	CuOTf	CH_2Cl_2	0	**65%**
**26**	**G**	CuOTf	Et_2O	5	**78%**
**27**	**J**	CuOTf	$CHCl_3$	1	**29%**
**28**	**H**	$CuNTf_2$	CH_2Cl_2	1	**53%**
**29**	**J**	$CuNTf_2$	Et_2O	0	**59%**
**30**	**G**	CuI	CH_2Cl_2	1	**42%**

3rd wave

Entry	Ligand	CuX	Solvent	TMSCl (equiv)	B(O*i*Pr)$_3$ (equiv)	ee
**31**	**G**	CuOTf	CH_2Cl_2	5	0	**60%**
**32**	**G**	CuOTf	CH_2Cl_2	1	0	**81%**
**33**	**G**	CuOTf	CH_2Cl_2	0	1	**44%**
**34**	**G**	$CuNTf_2$	Et_2O	0	0	**67%**
**35**	**G**	$CuNTf_2$	Et_2O	1	0	**73%**
**36**	**G**	$CuNTf_2$	Et_2O	5	0	**74%**
**37**	**G**	$CuNTf_2$	CH_2Cl_2	5	0	**72%**
**38**	**G**	$CuNTf_2$	CH_2Cl_2	0	1	**57%**

Conditions: 4-phenyl-1-butene (2.5 equiv), Cp_2ZrHCl (2.0 equiv), **2d** (1.0 equiv), Cu**L*** complex as specified (0.1 equiv), additive as specified (1.0 equiv), in specified solvent (2.0

mL), room temperature. [§]ee determined by HPLC. For more information on procedures see Supporting Information.

VI. Specific Procedures

3-(4-phenylbutyl)-3,6-dihydro-2*H*-pyran (5)

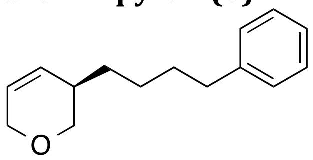

In a flame-dried flask under inert atmosphere, Cp_2ZrHCl (309 mg, 1.2 mmol, 2.0 equiv) was added to a solution of 4-phenyl-1-butene (220 μL, 1.5 mmol 2.5 equiv) in CH_2Cl_2 (0.6 mL) under an argon atmosphere and stirred vigorously until a clear yellow solution was obtained (20–40 min). Simultaneously, in another flask under inert atmosphere, CuCl (5.7 mg, 0.06 mmol, 0.1 equiv) and (*R*)-**D** (36.0 mg, 0.06 mmol, 0.1 equiv) were dissolved in CH_2Cl_2 (3.0 mL) and stirred for 1 h at room temperature. $AgClO_4$ (13.8 mg, 0.066 mmol, 0.11 equiv ***Perchlorates are explosive and should be handled with caution***) was added to the freshly formed Cu-ligand complex solution and stirred for 15 min. The resulting catalyst complex mixture was filtered into the freshly prepared alkylzirconocene species. After 10 min, 3-chloro-3,6-dihydro-2*H*-pyran (71 mg, 0.6 mmol, 1.0 equiv) was added dropwise via a microsyringe to the resulting black solution followed by the dropwise addition of $B(OiPr)_3$ (140 μL, 0.6 mmol, 1.0 equiv). The reaction mixture was stirred overnight. The reaction mixture was diluted with Et_2O (2 mL) and quenched with NH_4Cl (3 mL, 1 M). The mixture was partitioned and the aqueous phase was extracted with Et_2O (3 × 10 mL). The combined organic extracts were washed with $NaHCO_3$ (aq., sat., 30 mL), dried over $MgSO_4$, filtered and concentrated under reduced pressure. Purification by flash column chromatography (SiO_2, Et_2O 0➔2% in pentane) yielded the title product as a colourless oil (30.2 mg, 0.14 mmol, 25% yield, 100% conversion, 83% ee).

In a flame-dried flask under inert atmosphere, Cp_2ZrHCl (309 mg, 1.2 mmol, 2.0 equiv) was added to a solution of 4-phenyl-1-butene (220 μL, 1.5 mmol, 2.5 equiv) in CH_2Cl_2 (0.6 mL) under an argon atmosphere and stirred vigorously until a clear yellow solution was obtained (20–40 min). Simultaneously, in another flask under inert atmosphere, CuCl (5.7 mg, 0.06 mmol, 0.1 equiv) and (*S, S*)-**A** (29.7 mg, 0.06 mmol, 0.1 equiv) were dissolved in CH_2Cl_2 (3.0 mL) and stirred for 1 h at room temperature. AgOTf (17.1 mg, 0.066 mmol, 0.11 equiv) was added to the freshly formed Cu-ligand complex solution and stirred for 15 min. The resulting catalyst complex mixture was filtered into the freshly prepared alkylzirconocene species. After 10 min, 3,6-dihydro-2*H*-pyran-3-yl diethyl phosphate (142 mg, 0.6 mmol, 1.0 equiv) was added dropwise via a microsyringe to the resulting black solution and stirred overnight. The reaction mixture was diluted with Et_2O (2 mL) and quenched with NH_4Cl (3 mL, 1 M). The mixture was partitioned and the aqueous phase was extracted with Et_2O (3 × 10 mL). The combined organic extracts were washed with $NaHCO_3$ (aq., sat., 30 mL), dried over $MgSO_4$, filtered and concentrated under reduced

pressure. Purification by flash column chromatography (SiO_2, Et_2O 0→2% in pentane) yielded the title product as a colourless oil (22.5 mg, 0.10 mmol, 17% yield, 31% conversion, 83% ee).

^{1}H NMR (400 MHz, $CDCl_3$) δ_H /ppm 7.36-7.30 (m, 2H), 7.26-7.21 (m, 3H), 5.82 (ddd, 10.4 Hz, 4.7 Hz, 2.2 Hz, 1H), 5.75 (ddd, 10.1 Hz, 4.2 Hz, 2.2 Hz, 1H), 4.17-4.13 (m, 2H), 3.90 (dd, 10.9 Hz, 4.7 Hz, 1H), 3.45 (dd, 11.1 Hz, 6.7 Hz, 1H), 2.67 (t, 7.7 Hz, 2H), 2.28-2.17 (m, 1H), 1.73-1.62 (m, 2H), 1.46-1.35 (m, 4H).

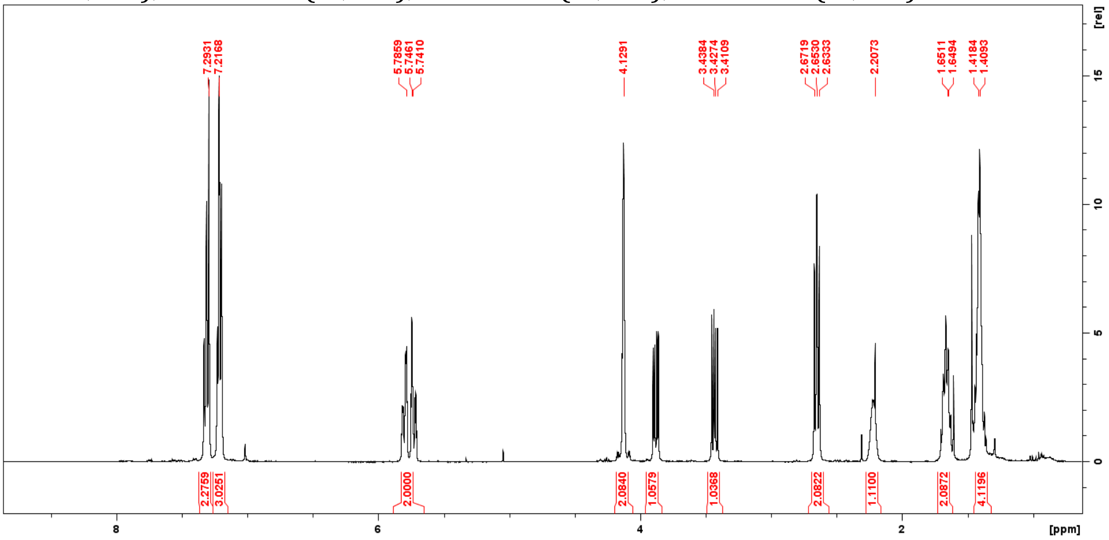

^{13}C NMR (100 MHz, $CDCl_3$) δ_C /ppm 142.6, 129.3, 128.4, 128.3, 125.8, 125.7, 69.3, 65.6, 35.9, 34.6, 32.4, 31.7, 26.5.

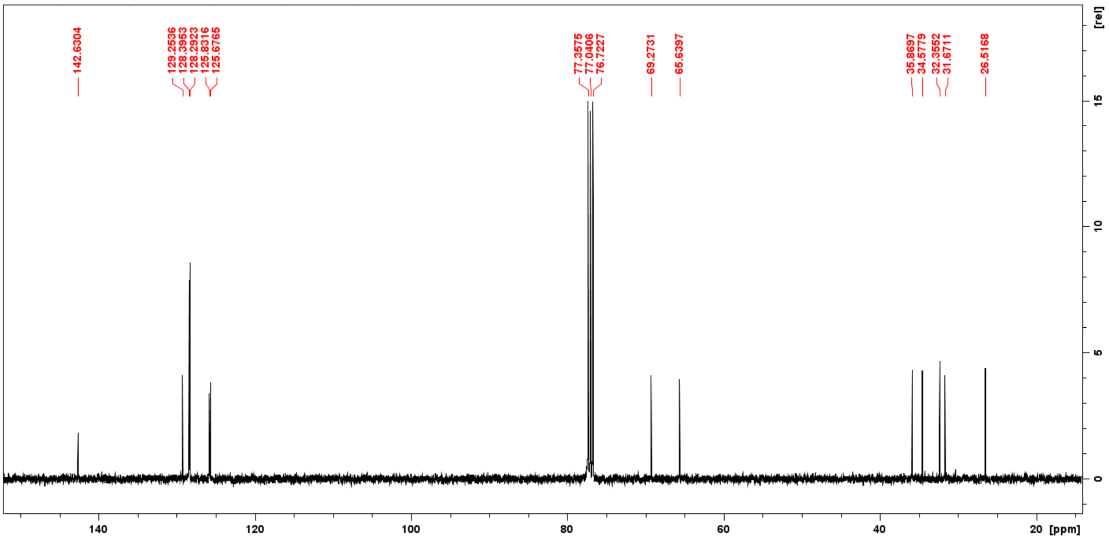

HRMS (CI GCMS) *m*/z calcd for $C_{15}H_{20}O$ $[M+H]^+$: 217.1587, found: 217.1586.

IR (ATR) (ν_{max}/cm^{-1}) 3027, 2929, 2856, 1496, 1454, 1117, 1087, 746, 699;

$[\alpha]^{20}{}_{589}$ = -46.3 (c= 0.97 in $CHCl_3$, 80% ee). (From phosphate)

The enantiomeric excess of 83% was determined by HPLC [Chiralpak® IB; hexane:*i*PrOH 99.2:0.8; 1.0 ml.min^{-1}, λ= 210 nm, t_R = 5.67 min (minor enantiomer), t_R = 6.07 min (major enantiomer)].

From phosphate (83% ee) (*R*)- 3-(4-phenylbutyl)-3,6-dihydro-2*H*-pyran

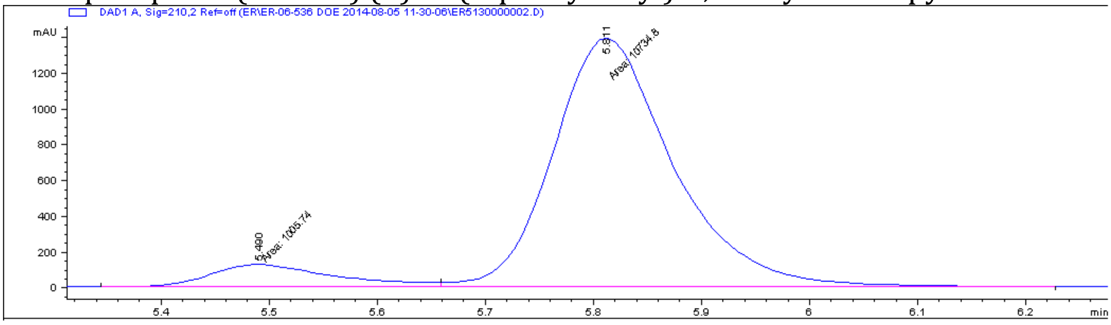

From chloride (83% ee) (*S*)- 3-(4-phenylbutyl)-3,6-dihydro-2*H*-pyran

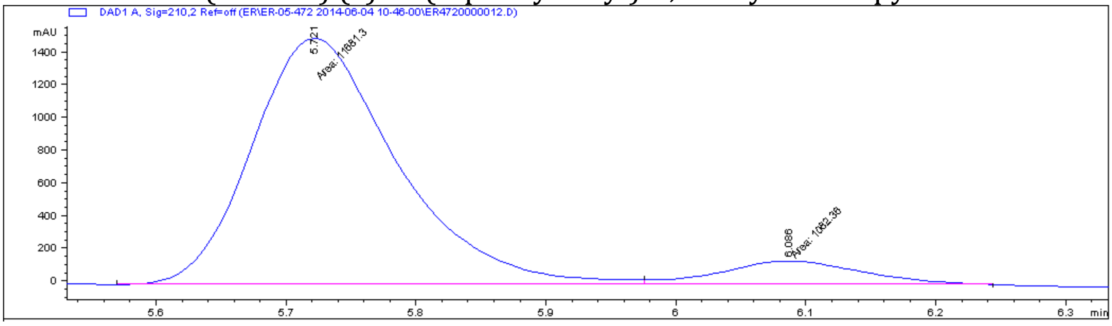

Racemic

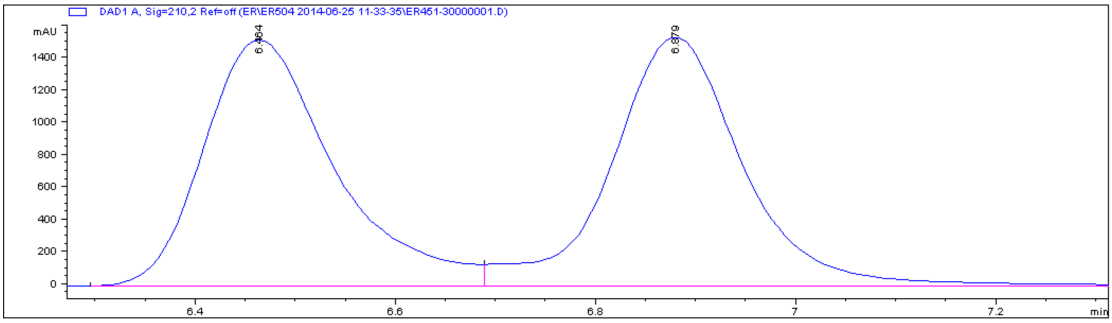

3-(3-(4-(trifluoromethyl)phenyl)propyl)-3,6-dihydro-2*H*-pyran (6)

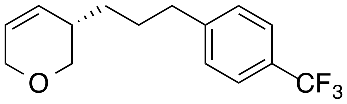

In a flame-dried flask under inert atmosphere, Cp_2ZrHCl (309 mg, 1.2 mmol, 2.0 equiv) was added to a solution of 1-allyl-4-(trifluoromethyl)benzene (250 μL, 1.5 mmol 2.5 equiv) in CH_2Cl_2 (0.6 mL) under an argon atmosphere and stirred vigorously until a clear yellow solution was obtained (20–40 min). Simultaneously, in another flask under inert atmosphere, CuCl (5.7 mg, 0.06 mmol, 0.1 equiv) and (*R*)-**D** (36.0 mg, 0.06 mmol, 0.1 equiv) were dissolved in CH_2Cl_2 (3.0 mL) and stirred for 1 h at room temperature. $AgClO_4$ (13.8 mg, 0.066 mmol, 0.11 equiv ***Perchlorates are explosive and should be handled with caution***) was added to the freshly formed Cu-ligand complex solution and stirred for 15 min. The resulting catalyst complex mixture was filtered into the freshly

prepared alkylzirconocene species. After 10 min, 3-chloro-3,6-dihydro-2*H*-pyran (71 mg, 0.6 mmol, 1.0 equiv) was added dropwise via a microsyringe to the resulting black solution followed by the dropwise addition of $B(OiPr)_3$ (140 μL, 0.6 mmol, 1.0 equiv). The reaction mixture was stirred overnight. The reaction mixture was diluted with Et_2O (2 mL) and quenched with NH_4Cl (3 mL, 1 M). The mixture was partitioned and the aqueous phase was extracted with Et_2O (3 × 10 mL). The combined organic extracts were washed with $NaHCO_3$ (aq., sat., 30 mL), dried over $MgSO_4$, filtered and concentrated under reduced pressure. Purification by flash column chromatography (SiO_2, Et_2O 0➔2% in pentane) yielded the title product as a colourless oil (28.5 mg, 0.11 mmol, 18% yield, 75% ee)

^{1}H NMR (400 MHz, $CDCl_3$) δ_H/ppm 7.54 (d, 8.3 Hz, 2H), 7.29 (d, 7.9 Hz, 2H), 5.82-5.69 (m, 2H), 4.16-4.05 (m, 2H), 3.86 (dd, 11.4 Hz, 4.8 Hz, 1H), 3.43 (dd, 11.4 Hz, 6.5Hz, 1H), 2.68 (t, 7.6 Hz,, 2H), 2.27-2.17 (m, 1H), 1.76-1.65 (m, 2H), 1.45-1.35 (m, 2H).

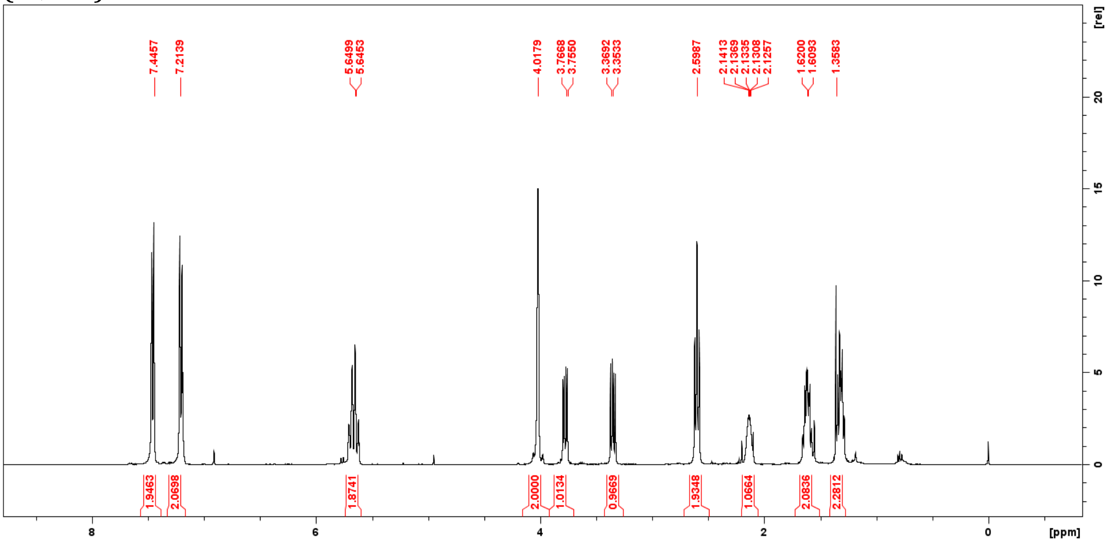

^{13}C NMR (100 MHz, $CDCl_3$) δ_C/ppm 146.4, 129.3, 128.8, 128.7, 126.2, 125.3, 125.2, 69.1, 65.6, 35.9, 34.5, 32.1, 28.4.

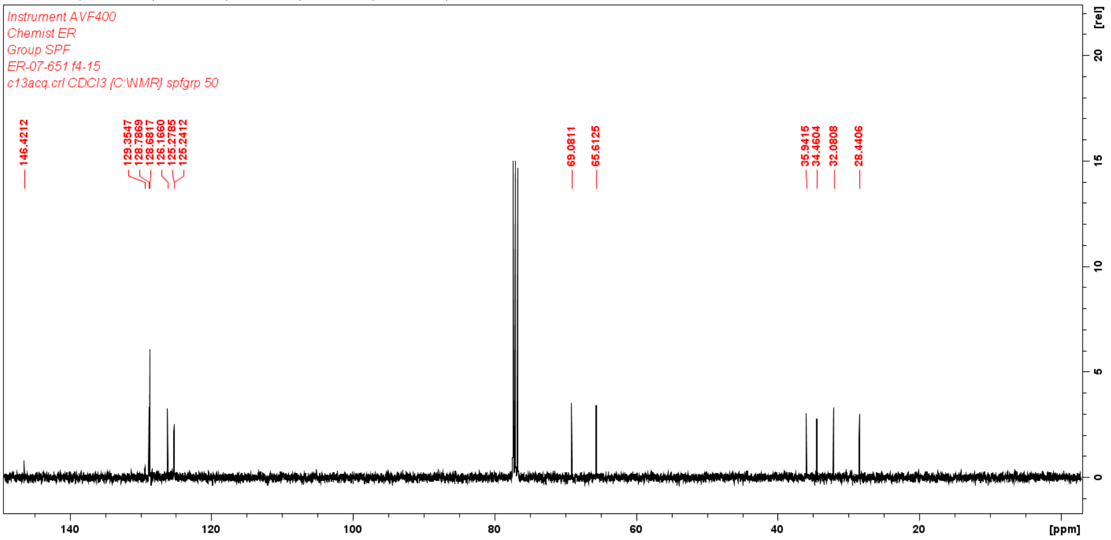

^{19}F NMR (377 MHz, $CDCl_3$) δ_F/ppm -62.28 (1F, s).

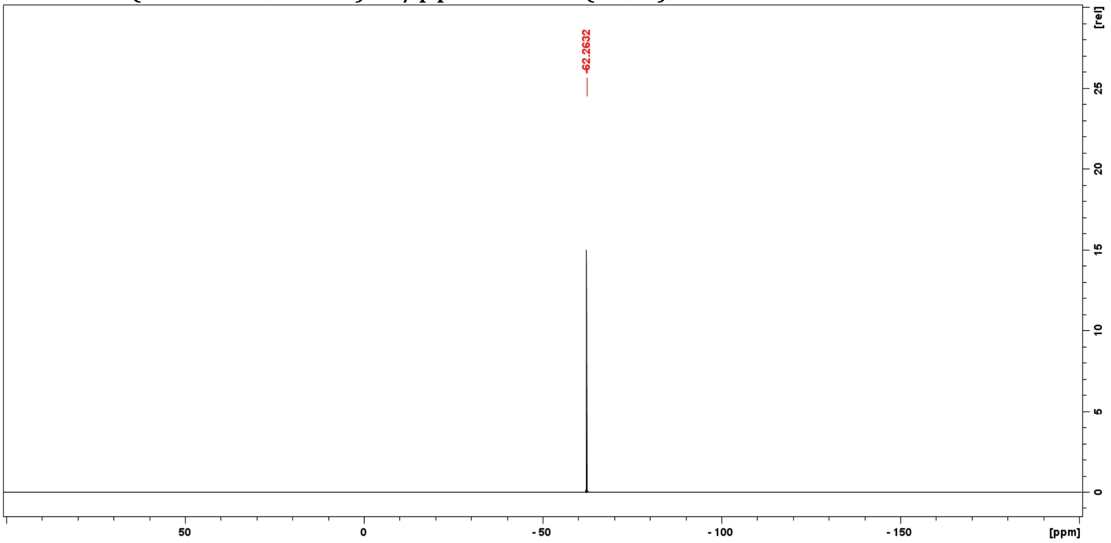

HRMS (EI/FI) *m*/z calcd for $C_{15}H_{17}OF_3$ $[M]^+$:270.1231, found:270.1232.

IR (ATR) (ν_{max}/cm^{-1}) 2933, 1326, 1164, 1122, 1068, 1019, 908, 733.

$[\alpha]^{20}$ $_{589}$ = +30.6 (c=0.78 in $CHCl_3$, 75% ee).

GC analysis of the crude mixture of epoxides derived from **6** indicated an enantiomeric excess of 75% (Hydrodex 6-TBDM, 60–170 ^oC at 1 ^oC/min, 170 ^oC for 100 min, 10 psi); major enantiomer t_R= 135.5, 149.8 min; minor enantiomer t_R = 133.4, 164.8 min.

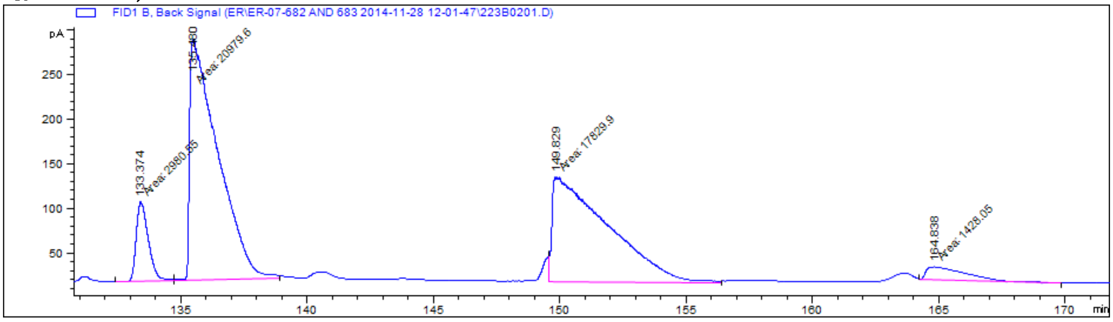

Racemic

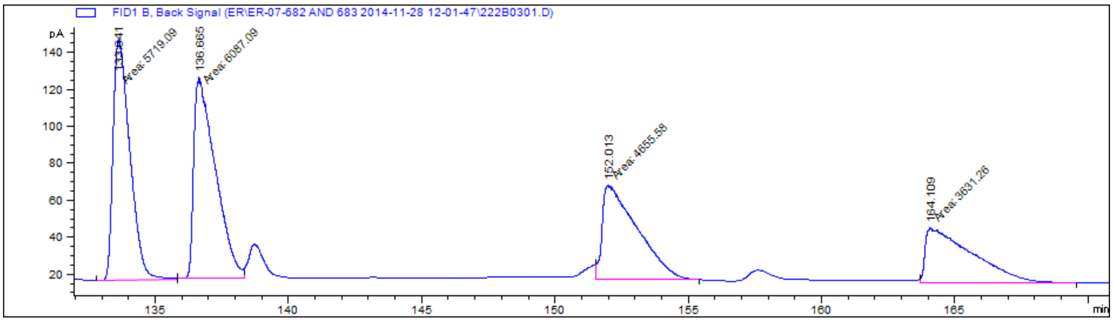

3-(6-chlorohexyl)-3,6-dihydro-2*H*-pyran (7)

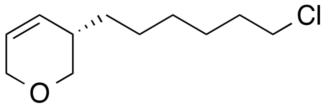

In a flame-dried flask under inert atmosphere, Cp_2ZrHCl (309 mg, 1.2 mmol, 2.0 equiv) was added to a solution of 6-chlorohex-1-ene (200 μL, 1.5 mmol 2.5 equiv) in CH_2Cl_2 (0.6 mL) under an argon atmosphere and stirred vigorously until a clear yellow solution was obtained (20-40 min). Simultaneously, in another flask under inert atmosphere, CuCl (5.7 mg, 0.06 mmol, 0.1 equiv) and (*R*)-**D** (36.0 mg, 0.06 mmol, 0.1 equiv) were dissolved in CH_2Cl_2 (3.0 mL) and stirred for 1 h at room temperature. $AgClO_4$ (13.8 mg, 0.066 mmol, 0.11 eq ***Perchlorates are explosive and should be handled with caution***) was added to the freshly formed Cu-ligand complex solution and stirred for 15 min. The resulting catalyst complex mixture was filtered into the freshly prepared alkylzirconocene species. After 10 min, 3-chloro-3,6-dihydro-2*H*-pyran (71 mg, 0.6 mmol, 1.0 equiv) was added dropwise via a microsyringe to the resulting black solution followed by the dropwise addition of $B(OiPr)_3$ (140 μL, 0.6 mmol, 1.0 equiv). The reaction mixture was stirred overnight. The reaction mixture was diluted with Et_2O (2 mL) and quenched with NH_4Cl (3 mL, 1M). The mixture was partitioned and the aqueous phase was extracted with Et_2O (3 × 10 mL). The combined organic extracts were washed with $NaHCO_3$ (aq., sat., 30 mL), dried over $MgSO_4$, filtered and concentrated under reduced pressure. Purification by flash column chromatography (SiO_2, Et_2O 0➔2% in pentane) yielded the title product as a colourless oil (40.5 mg, 0.20 mmol, 33% yield, 77% ee).

^{1}H NMR (400 MHz, $CDCl_3$) δ_H /ppm 5.80-5.73 (m, 1H), 5.73-5.67 (m, 1H), 4.16-4.05 (m, 2H), 3.87 (dd, 10.9 Hz, 6.6 Hz, 1H), 3.54 (t, 6.8 Hz, 2H), 3.41 (dd, 11.1 Hz, 4.9 Hz, 1H), 2.24-2.15 (m, 1H), 1.78 (tt, 14.8 Hz, 6.9 Hz, 2H), 1.50-1.40 (m, 2H), 1.39-1.30 (m, 6H).

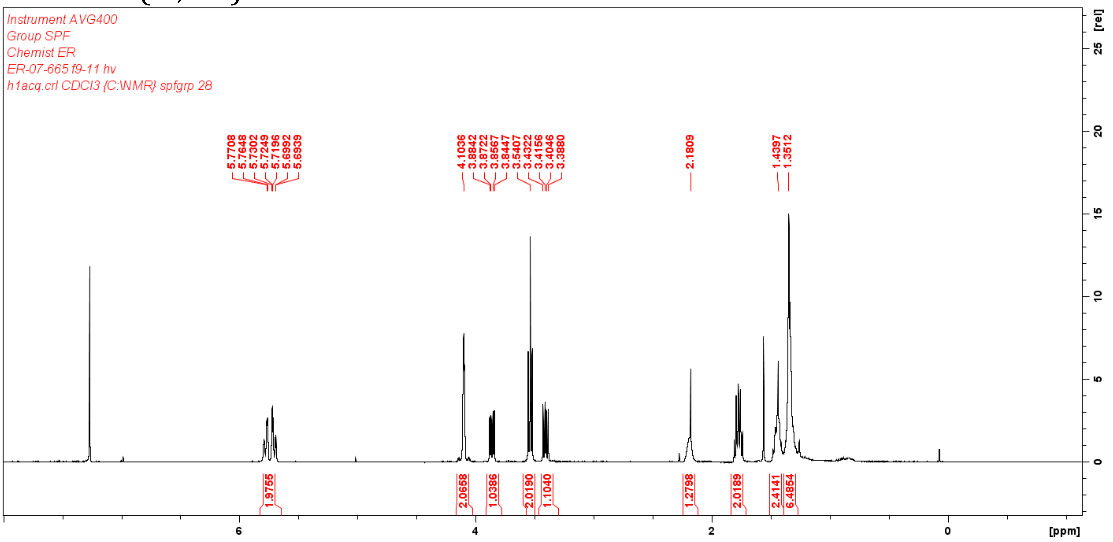

^{13}C NMR (100 MHz, $CDCl_3$) δ_C /ppm 129.2, 125.8, 69.3, 65.6, 45.1, 34.6, 32.6, 32.4, 29.1, 26.8, 26.7.

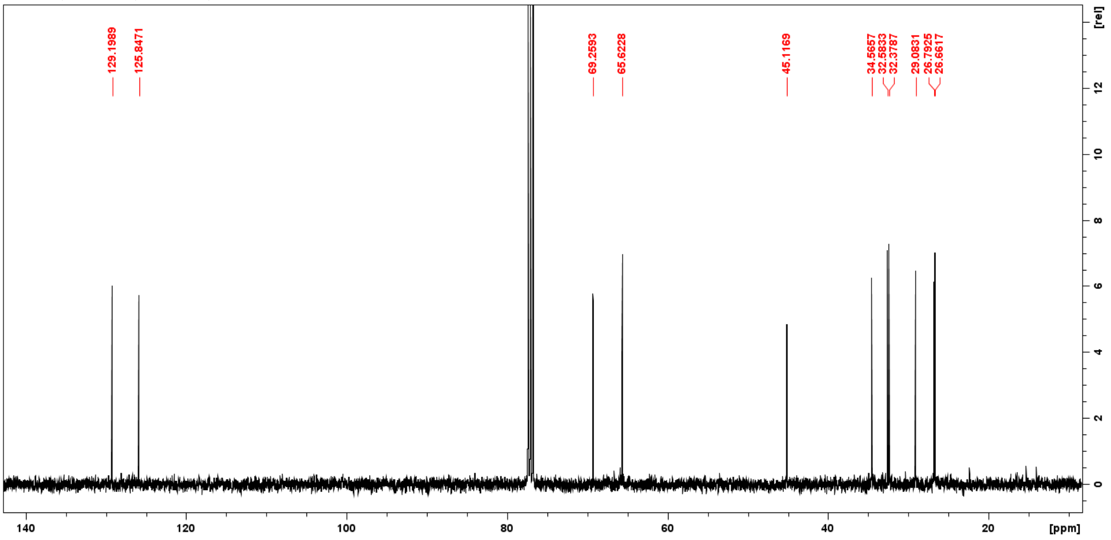

HRMS (ESI) *m*/z calcd for $C_{11}H_{19}OClNa$ $[M+Na]^+$:225.10166, found:225.10132.

IR (ATR) (ν_{max}/cm^{-1}) 2928, 2855, 1458, 1142, 1088.

$[\alpha]^{20}{}_{589}$ = +46.6 (c=1.11 in $CDCl_3$, 77% ee)

GC analysis of the crude mixture of epoxides derived from **7** indicated an enantiomeric excess of 77% (Hydrodex 6-TBDM, 60–170 °C at 1 °C/min, 170 °C for 70 min, 10 psi); major enantiomer t_R= 114.8, 122.9 min; minor enantiomer t_R = 115.9, 126.2 min.

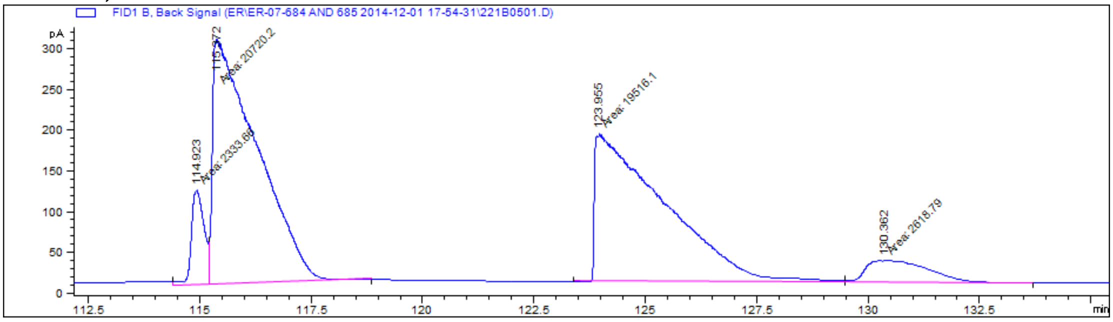

Racemic

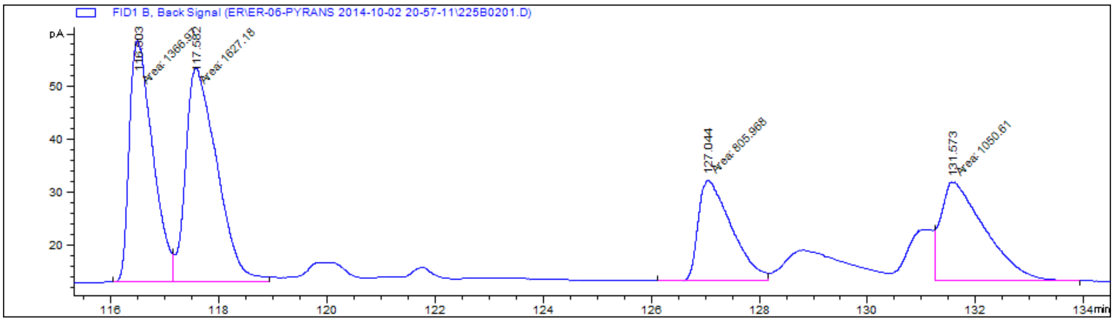

3-(2-cyclohexylethyl)-3,6-dihydro-2*H*-pyran (8)

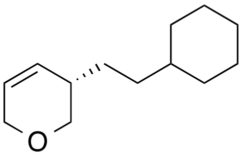

In a flame-dried flask under inert atmosphere, Cp_2ZrHCl (309 mg, 1.2 mmol, 2.0 equiv) was added to a solution of vinylcyclohexane (210 μL, 1.5 mmol 2.5 equiv) in CH_2Cl_2 (0.6 mL) under an argon atmosphere and stirred vigorously until a clear yellow solution was obtained (20-40 min). Simultaneously, in another flask under inert atmosphere, CuCl (5.7 mg, 0.06 mmol, 0.1 equiv) and (*R*)-**D** (36.0 mg, 0.06 mmol, 0.1 equiv) were dissolved in CH_2Cl_2 (3.0 mL) and stirred for 1 h at room temperature. $AgClO_4$ (13.8 mg, 0.066 mmol, 0.11 equiv ***Perchlorates are explosive and should be handled with caution***) was added to the freshly formed Cu-ligand complex solution and stirred for 15 min. The resulting catalyst complex mixture was filtered into the freshly prepared alkylzirconocene species. After 10 min, 3-chloro-3,6-dihydro-2*H*-pyran (71 mg, 0.6 mmol, 1.0 equiv) was added dropwise via a microsyringe to the resulting black solution followed by the dropwise addition of B(O*i*Pr)$_3$ (140 μL, 0.6 mmol, 1.0 equiv). The reaction mixture was stirred overnight. The reaction mixture was diluted with Et_2O (2 mL) and quenched with NH_4Cl (3 mL, 1 M). The mixture was partitioned and the aqueous phase was extracted with Et_2O (3 × 10 mL). The combined organic extracts were washed with $NaHCO_3$ (aq., sat., 30 mL), dried over $MgSO_4$, filtered and concentrated under reduced pressure. Purification by flash column chromatography (SiO_2, Et_2O 0➔2% in pentane) yielded the title product as a colourless oil (23.8 mg, 0.12 mmol, 20% yield, 88% ee)

^{1}H NMR (400 MHz, $CDCl_3$) δ_H/ppm 5.71 (ddd, 10.3 Hz, 5.5 Hz, 2.3 Hz, 1H), 5.62 (ddd, 10.3 Hz, 5.2 Hz, 2.2 Hz, 1H), 4.04-4.00 (m, 2H), 3.79 (ddd, 11.0 Hz, 4.8 Hz, 0.4 Hz, 1H), 3.31 (dd, 11.3 Hz, 6.9 Hz, 1H), 2.14-2.03 (m, 1H) 1.69-1.52 (m, 4H), 1.31-1.06 (m, 9H), 0.88-0.73 (m, 2H).

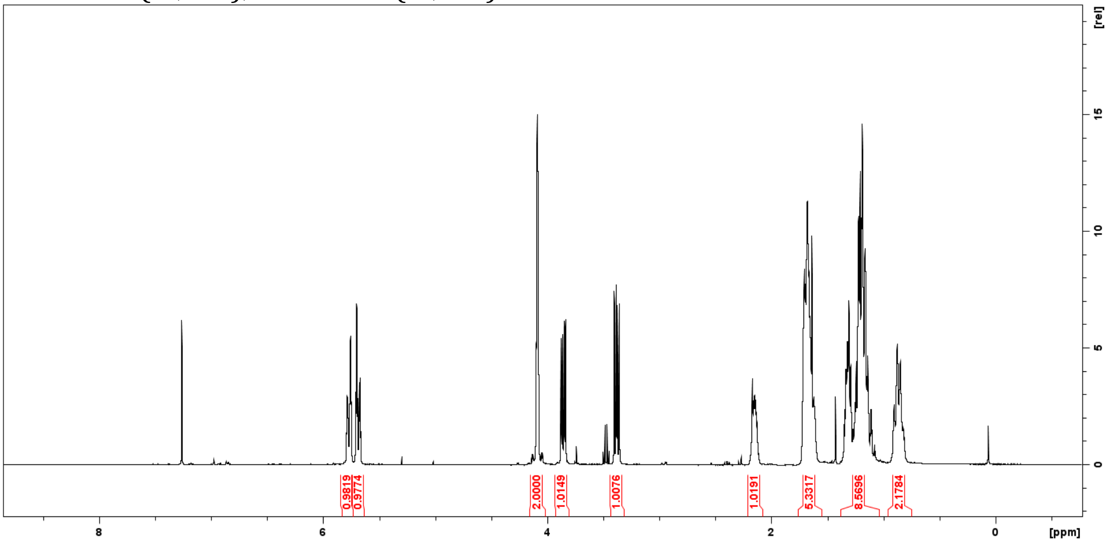

^{13}C NMR (100 MHz, $CDCl_3$) δ_C /ppm 129.4, 125.7, 69.4, 65.6, 37.9, 34.9, 34.5, 33.4, 29.8, 26.7, 26.4.

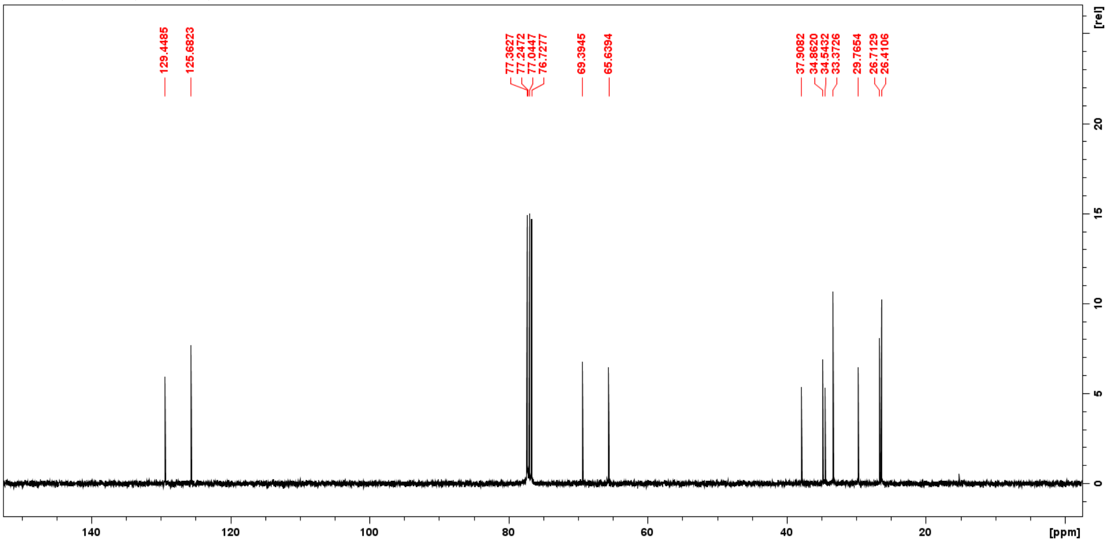

HRMS (CI GCMS) *m*/z calcd for $[M]^+$: 194.1671, found: 194.1668.

IR (ATR) (ν_{max}/cm^{-1}) 3029, 2919, 2850, 1448, 1224, 1089, 886, 701, 686.

$[\alpha]^{20}$ $_{589}$ = +55.6 (c= 1.07 in $CHCl_3$, 88% ee).

GC analysis of the crude mixture of epoxides derived from **8** indicated an enantiomeric excess of 88% (Hydrodex 6-TBDM, 60–165 °C at 1 °C/min, 165 °C for 70 min, 10 psi); major enantiomer t_R= 100.8, 105.2 min; minor enantiomer t_R = 100.2, 109.1 min.

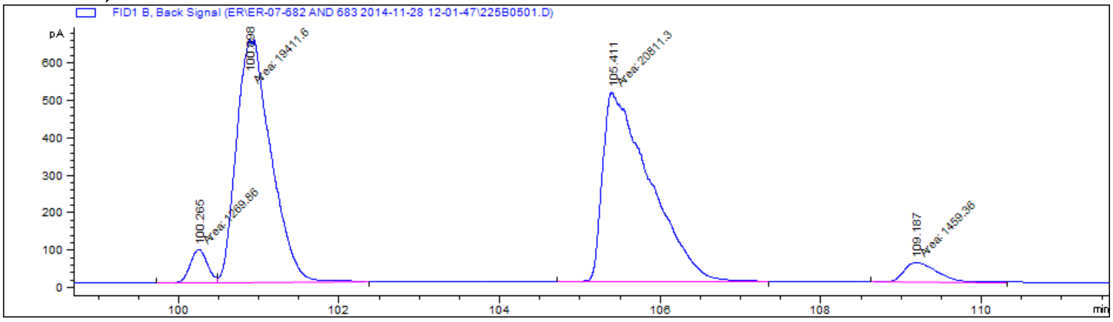

Racemic

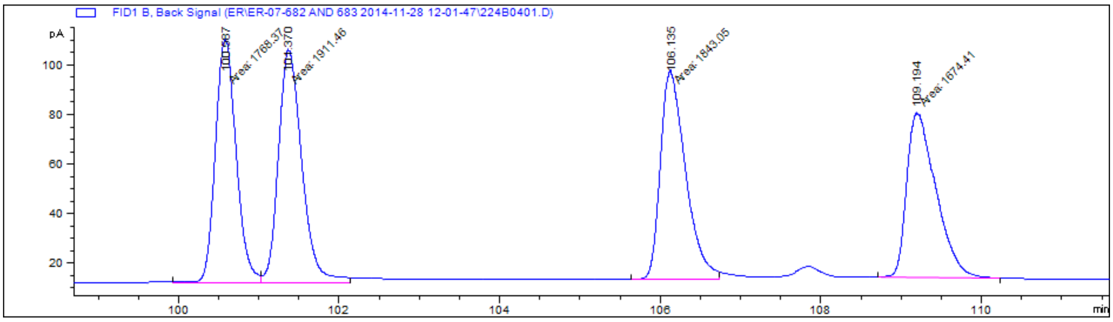

(3-(3,6-dihydro-2*H*-pyran-3-yl)propyl)trimethylsilane (9)

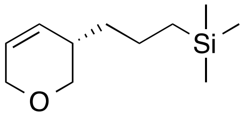

In a flame-dried flask under inert atmosphere, Cp_2ZrHCl (412 mg, 1.6 mmol, 2.0 equiv) was added to a solution of allyltrimethylsilane (320 μL, 2.0 mmol 2.5 equiv) in CH_2Cl_2 (0.8 mL) under an argon atmosphere and stirred vigorously until a clear yellow solution was obtained (20-40 min). Simultaneously, in another flask under inert atmosphere, CuCl (7.6 mg, 0.08 mmol, 0.1 equiv) and (*R*)-**D** (48.0 mg, 0.08 mmol, 0.1 equiv) were dissolved in CH_2Cl_2 (4.0 mL) and stirred for 1 h at room temperature. $AgClO_4$ (18.2 mg, 0.088 mmol, 0.11 equiv ***Perchlorates are explosive and should be handled with caution***) was added to the freshly formed Cu-ligand complex solution and stirred for 15 min. The resulting catalyst complex mixture was filtered into the freshly prepared alkylzirconocene species. After 10 min, 3-chloro-3,6-dihydro-2*H*-pyran (95 mg, 0.8 mmol, 1.0 equiv) was added dropwise via a microsyringe to the resulting black solution followed by the dropwise addition of $B(OiPr)_3$ (180 μL, 0.8 mmol, 1.0 equiv). The reaction mixture was stirred overnight. The reaction mixture was diluted with Et_2O (2 mL) and quenched with NH_4Cl (3 mL, 1 M). The mixture was partitioned and the aqueous phase was extracted with Et_2O (3 × 10 mL). The combined organic extracts were washed with $NaHCO_3$ (aq., sat., 30 mL), dried over $MgSO_4$, filtered and concentrated under reduced pressure. Purification by flash column chromatography (SiO_2, Et_2O 0→2% in pentane) yielded the title product as a volatile colourless oil (13.1 mg, 0.066 mmol, 8% yield, 93% ee).

^{1}H NMR (400 MHz, $CDCl_3$) δ_H/ppm 5.77(ddd, 10.3, 4.5, 2.1 Hz, 1H), 5.69 (ddd, 10.3, 4.5, 2.2 Hz, 1H), 4.13-4.08 (m, 2H), 3.86 (dd, 10.9, 4.8 Hz, 1H), 3.39 (dd, 10.9, 6.9 Hz, 1H), 2.26-2.18 (br. m, 1H), 1.38-1.32 (m, 4H), 0.53-0.45 (m, 2H), -0.02 (s, 9H).

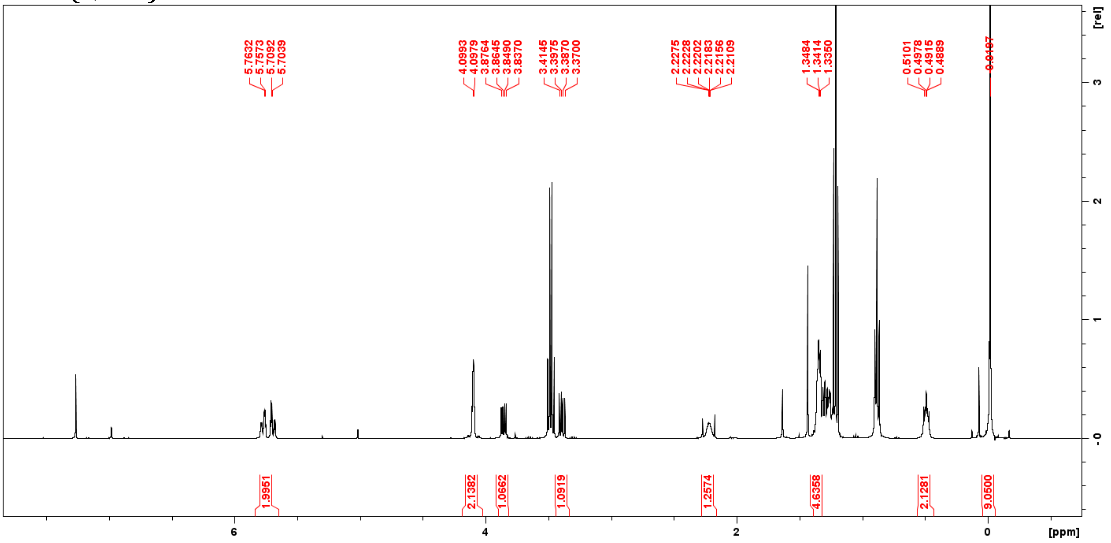

^{13}C NMR (100 MHz, $CDCl_3$) δ_C/ppm 129.5, 125.8, 69.4, 65.8, 21.3, 17.0, -1.5.

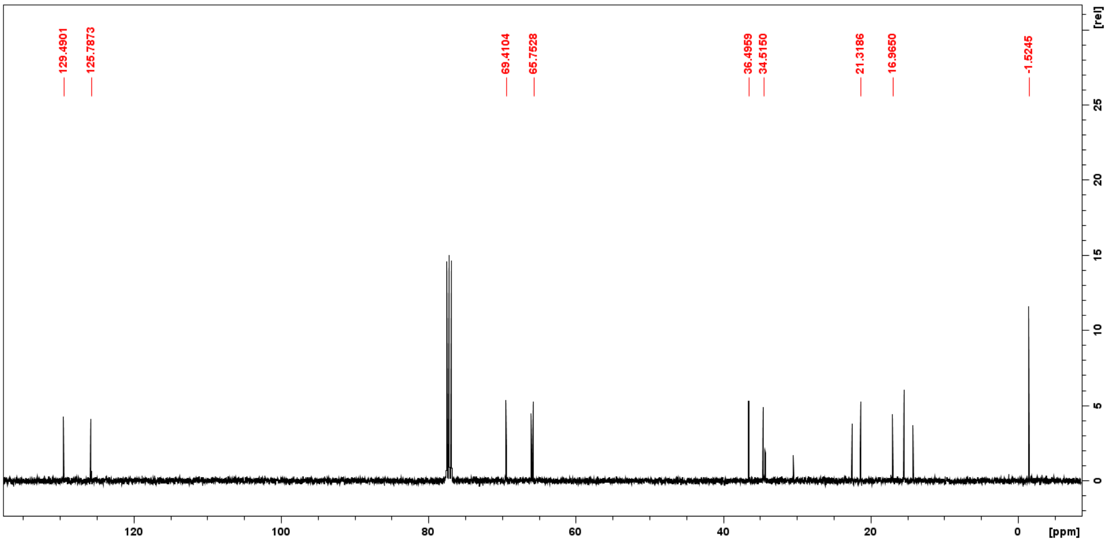

HRMS (EI/FI) *m*/z calcd for $C_{11}H_{22}OSi$ $[M]^+$: 198.1440, found: 198.1446.

IR (ATR) (ν_{max}/cm^{-1}) 2953, 2920, 2856, 1248, 1109, 1089, 861, 836.

$[\alpha]^{20}{}_{589}$ = +33.3 (c=1.01 in $CDCl_3$, 93% ee).

GC analysis of the crude mixture of epoxides derived from **9** indicated an enantiomeric excess of 93% (Hydrodex 6-TBDM, 60–170 °C at 1 °C/min, 170 °C for 50 min, 10 psi); major enantiomer t_R= 76.6, 81.6 min; minor enantiomer t_R = 75.7, 86.2 min.

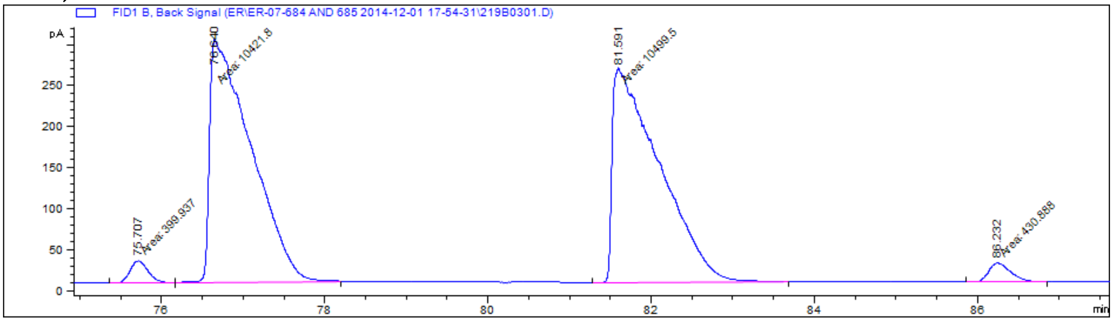

Racemic

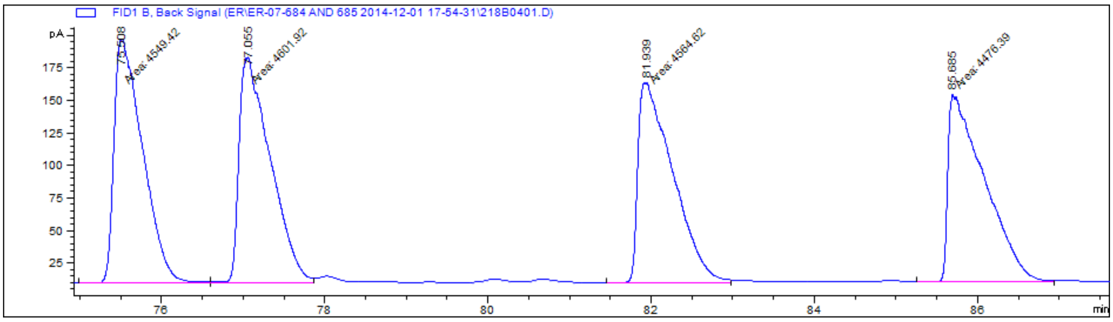

VII. Side-product

3,3',6,6'-tetrahydro-2*H*,2'*H*-3,3'-bipyran (11)

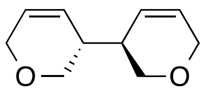

In a flame-dried flask under inert atmosphere, Cp_2ZrHCl (309 mg, 1.2 mmol, 2.0 equiv) was added to a solution of 4-phenyl-1-butene (230 μL, 1.5 mmol 2.5 equiv) in CH_2Cl_2 (0.6 mL) under an argon atmosphere and stirred vigorously until a clear yellow solution was obtained (20-40 min). Simultaneously, in another flask under inert atmosphere, CuCl (5.7 mg, 0.06 mmol, 0.1 equiv) and (*R*)-**D** (36.0 mg, 0.06 mmol, 0.1 equiv) were dissolved in CH_2Cl_2 (3.0 mL) and stirred for 1 h at room temperature. $AgClO_4$ (13.8 mg, 0.066 mmol, 0.11 equiv ***Perchlorates are explosive and should be handled with caution***) was added to the freshly formed Cu-ligand complex solution and stirred for 15 min. The resulting catalyst complex mixture was filtered into the freshly prepared alkylzirconocene species. After 10 min, 3-chloro-3,6-dihydro-2*H*-pyran (71 mg, 0.6 mmol, 1.0 equiv) was added dropwise via a microsyringe to the resulting black solution followed by the dropwise addition of $B(O\textit{i}Pr)_3$ (140 μL, 0.6 mmol, 1.0 equiv). The reaction mixture was stirred overnight. The reaction mixture was diluted with Et_2O (2 mL) and quenched with NH_4Cl (3 mL, 1 M). The mixture was partitioned and the aqueous phase was extracted with Et_2O (3 × 10 mL). The combined organic extracts were washed with $NaHCO_3$ (aq., sat., 30 mL), dried over $MgSO_4$, filtered and concentrated under reduced pressure. Purification by flash column chromatography (SiO_2, Et_2O 0→2% in pentane) yielded the enantioenriched product as a colourless oil (30.2 mg, 0.14 mmol, 23%) and the title dimer side-product (SiO_2, Et_2O 20% in pentane) as a yellow oil (30.0 mg, 0.36 mmol, 60% yield)

^{1}H NMR (400 MHz, $CDCl_3$) δ_H /ppm 5.84-5.75 (m, 4H), 4.12-4.06 (m, 4H), 3.84 (dd, *J*= 11.5, 4.3 Hz, 1H), 3.75(dd, *J*= 11.5, 4.4 Hz, 1H), 3.68 (dd, *J*= 11.2, 4.7 Hz, 1H), 3.64 (dd, *J*= 11.5, 5.6 Hz, 1H), 2.35-2.25 (m, 2H).

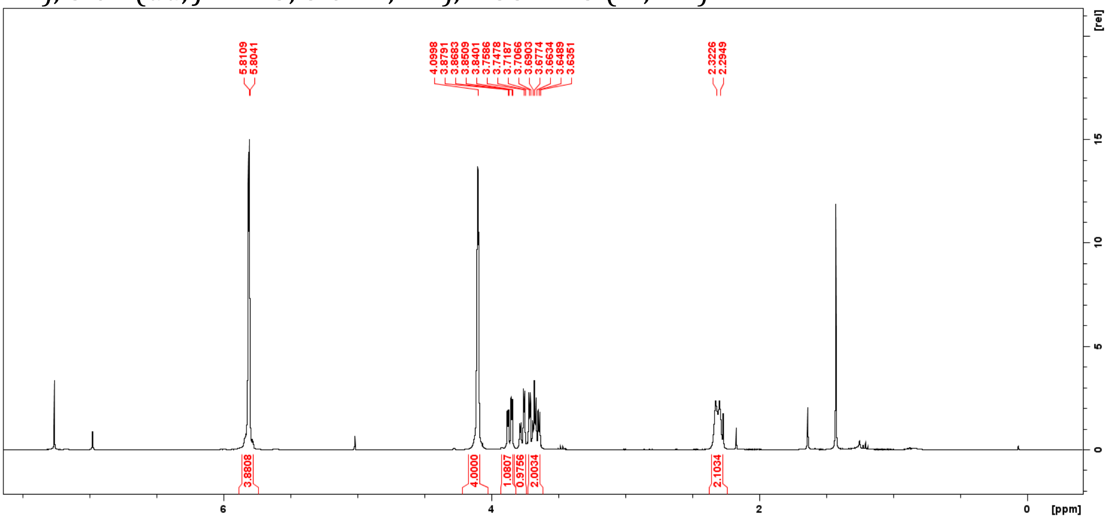

^{13}**C NMR** (100 MHz, $CDCl_3$) δ_C /ppm 127.7, 127.3, 126.8, 67.4, 67.0, 66.2, 65.8, 37.5, 37.2.

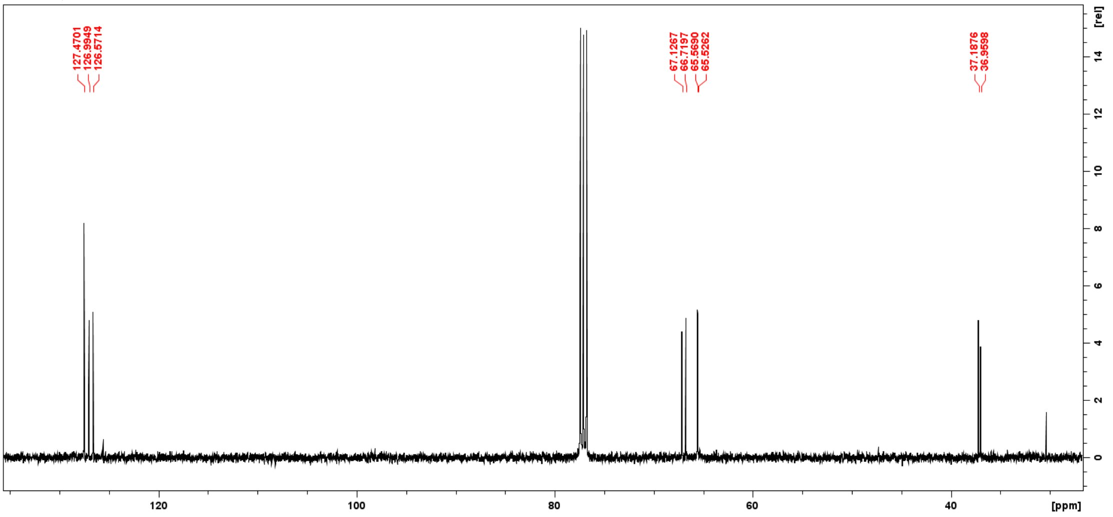

HRMS (ESI) *m*/z calcd for $C_{10}H_{15}O_2$ $[M+H]^+$: 167.10666, found: 167.10675.

IR (ATR) (ν_{max}/cm^{-1}) 3031, 2963, 2929, 2855, 2820, 1087, 1047, 1017.

$[\alpha]^{20}_{589}$ = -1.8 (c= 1.02 in $CHCl_3$).

Racemic synthesis of (3*R*,3'*R*)-3,3',6,6'-tetrahydro-2*H*,2'*H*-3,3'-bipyran (11)

In a flame-dried flask under inert atmosphere, Cp_2ZrHCl (206 mg, 0.7 mmol, 2.0 equiv) was added to a solution of alkene (150 μL, 1.0 mmol 2.5 equiv) in CH_2Cl_2 (0.4 mL) under an argon atmosphere and stirred vigorously until a clear yellow solution was obtained (20-40 min). Simultaneously, in another flask under inert atmosphere, CuCl (47 mg, 0.4 mmol, 0.1 equiv) and (*S, S, S*)-Feringa Ligand (10.8 mg, 0.02 mmol, 0.05 equiv) and (*R, R, R*)- Feringa Ligand (10.8 mg, 0.02 mmol, 0.05 equiv) were dissolved in CH_2Cl_2 (2.0 mL) and stirred for 1 h at room temperature. $AgClO_4$ (9.1 mg, 0.044 mmol, 0.11 equiv ***Perchlorates are explosive and should be handled with caution***) was added to the freshly formed Cu-ligand complex solution and stirred for 15 min. The resulting catalyst complex mixture was filtered into the freshly prepared alkylzirconocene species. After 10 min, 3-chloro-3,6-dihydro-2*H*-pyran (47 mg, 0.4 mmol, 1.0 equiv) was added dropwise via a microsyringe to the resulting black solution followed by the dropwise addition of $B(OiPr)_3$ (92 μL, 0.4 mmol, 1.0 equiv). The reaction mixture was stirred overnight. The reaction mixture was diluted with Et_2O (2 mL) and quenched with NH_4Cl (3 mL, 1 M). The mixture was partitioned and the aqueous phase was extracted with Et_2O (3 × 10 mL). The combined organic extracts were washed with $NaHCO_3$ (aq., sat., 30 mL), dried over $MgSO_4$, filtered and concentrated under reduced pressure. Purification by flash column chromatography (SiO_2, Et_2O 20% in pentane) yielded the racemic title product.

VIII. Mechanistic experiments

NMR experiments were carried out on Bruker AVB400 (400/100 MHz), DRX500 (500/125 MHz), AVB500 (500/125 MHz) or AVC500 (500/125 MHz) spectrometers. Processing was performed on Topspin 3.2. Kinetic NMR experiments were performed by pre-tuning, locking and shimming the NMR sample before adding the substrate. No re-locking and re-shimming was performed after the addition of the substrate to facilitate fast data collection. Kinetics integrations were calibrated on the assumption that at any t_n, the sum of starting material and products was 100%. All solvents were dried and distilled if necessary ($CDCl_3$) as well as degassed under a flow of argon for a minimum of 30 min. The solvents were stored at 0 °C, in a schlenk on molecular sieves, protected from light.

3-chloro-3,6-dihydro-2*H*-pyran (2a)
Kinetic NMR

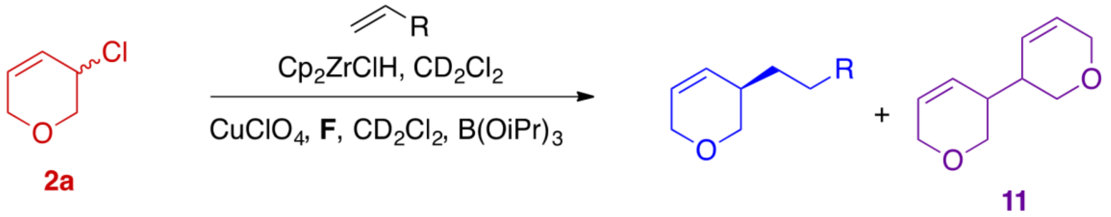

In a flame-dried flask under inert atmosphere, ethylene gas (1 atm) was bubble through a solution of Cp_2ZrHCl (103 mg, 0.4 mmol, 2.0 equiv) in CD_2Cl_2 (0.4 mL) under an argon atmosphere and stirred vigorously until a clear yellow solution was obtained (15 min). Simultaneously, in another flask under inert atmosphere, CuCl (1.9 mg, 0.02 mmol, 0.1 equiv) and (*R*)-**D** (12.0 mg, 0.02 mmol, 0.1 equiv) were dissolved in CD_2Cl_2 (1.0 mL) and stirred for 1 h at room temperature. $AgClO_4$ (4.6 mg, 0.022 mmol, 0.11 equiv ***Perchlorates are explosive and should be handled with caution***) was added to the freshly formed Cu-ligand complex solution and stirred for 15 min. The resulting catalyst complex mixture was filtered into a flamed dried NMR tube adapted with an NMR septa and parafilmed. Then the freshly prepared alkylzirconocene species was added to the NMR tube and the tube was thoroughly shaken to obtain an homogenous black solution. 3-chloro-3,6-dihydro-2*H*-pyran **2a** (24 mg, 0.2 mmol, 1.0 equiv) was added via a microsyringe to the NMR tube followed by $B(OiPr)_3$ (46 µL, 0.2 mmol, 1.0 equiv). The reaction mixture was shaken vigorously (upside-down mixing) and NMRs were recorded at regular intervals.

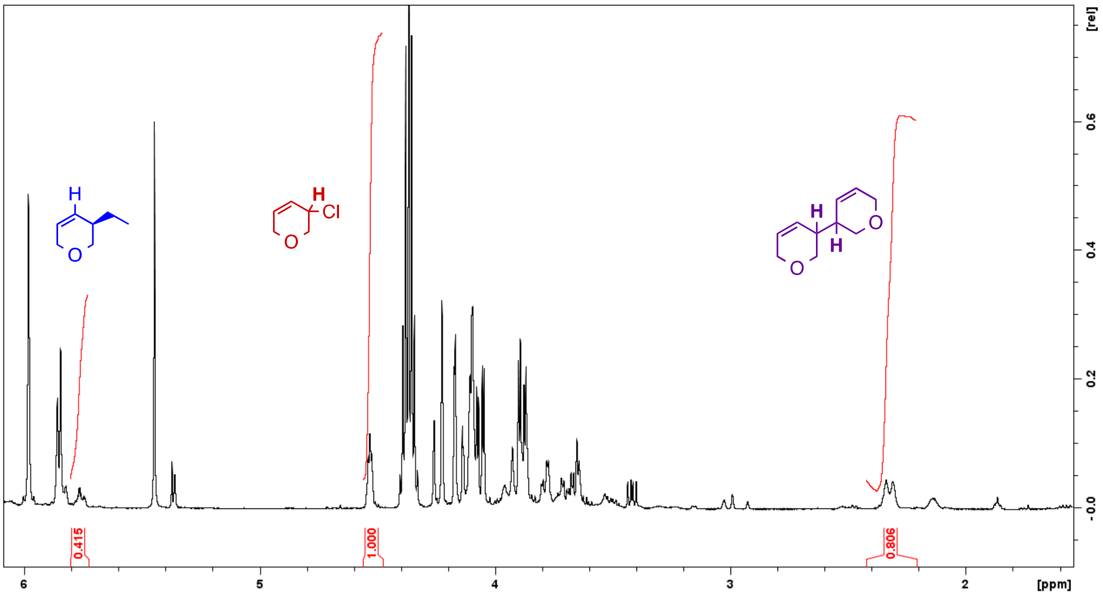

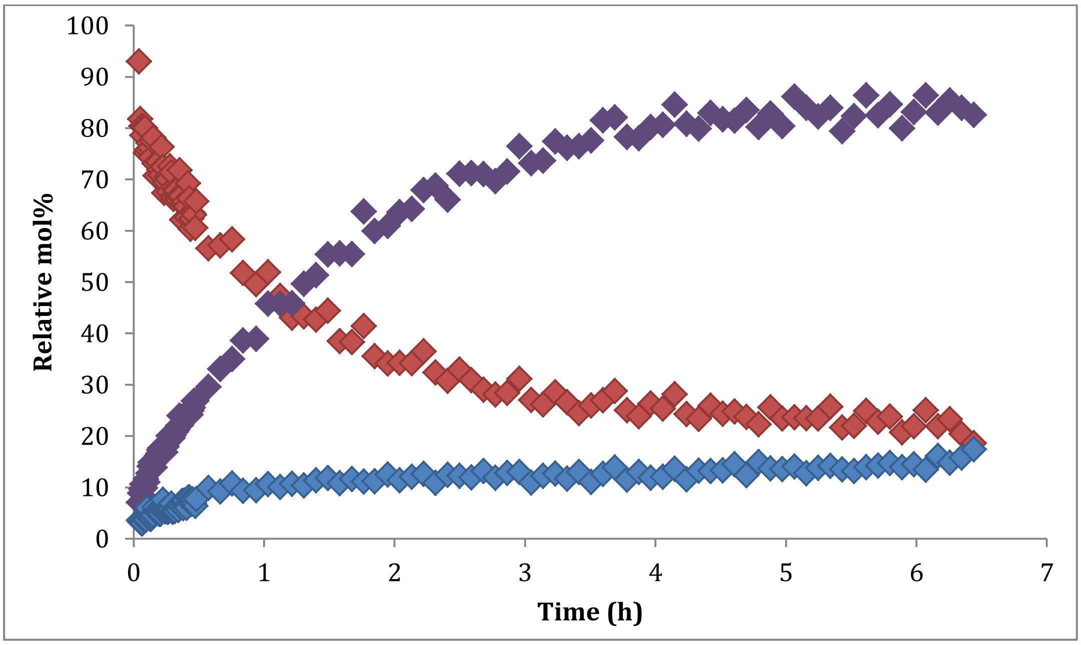

Kinetic ee

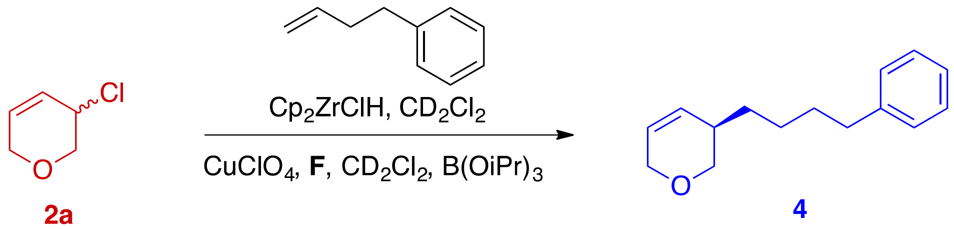

In a flame-dried flask under inert atmosphere, Cp_2ZrHCl (309 mg, 1.2 mmol, 2.0 equiv) was added to a solution of 4-phenyl-1-butene (230 μL, 1.5 mmol 2.5 equiv) in CH_2Cl_2 (0.6 mL) under an argon atmosphere and stirred vigorously until a clear yellow solution was obtained (20–40 min). Simultaneously, in another flask under inert atmosphere, CuCl (5.7 mg, 0.06 mmol, 0.1 equiv) and (*R*)- **D** (36.0 mg, 0.06 mmol, 0.1 equiv) were dissolved in

CH_2Cl_2 (3.0 mL) and stirred for 1 h at room temperature. $AgClO_4$ (13.8 mg, 0.066 mmol, 0.11 eq ***Perchlorates are explosive and should be handled with caution***) was added to the freshly formed Cu-ligand complex solution and stirred for 15 min. The resulting catalyst complex mixture was filtered into the freshly prepared alkylzirconocene species. After 10 min, 3-chloro-3,6-dihydro-2*H*-pyran **2a** (71 mg, 0.6 mmol, 1.0 equiv) was added dropwise via a microsyringe to the resulting black solution followed by the dropwise addition of $B(OiPr)_3$ (140 μL, 0.6 mmol, 1.0 equiv). The reaction mixture was stirred overnight. Aliquots were taken regularly and analysed by HPLC and GC to obtain the ee of product **5** and starting material **2a**.

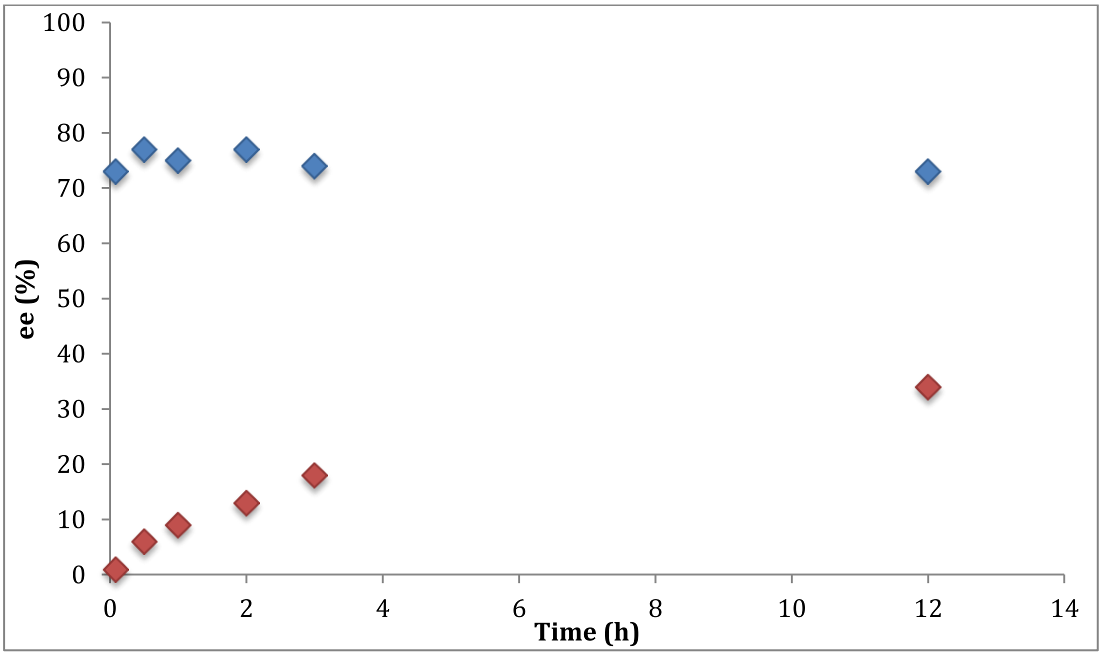

GC traces of 2a

The enantiomeric excess of 3-chloro-3,6-dihydro-2*H*-pyran **2a** was determined by GC [Hydrodex 6-TBDM, 60–120 °C at 1 °C/min, 10 psi, injector temperature 250 °C, detector temperature 300 °C; t_R= 34.8 min (major enantiomer), t_R = 39.0 min (minor enantiomer)].

5 min

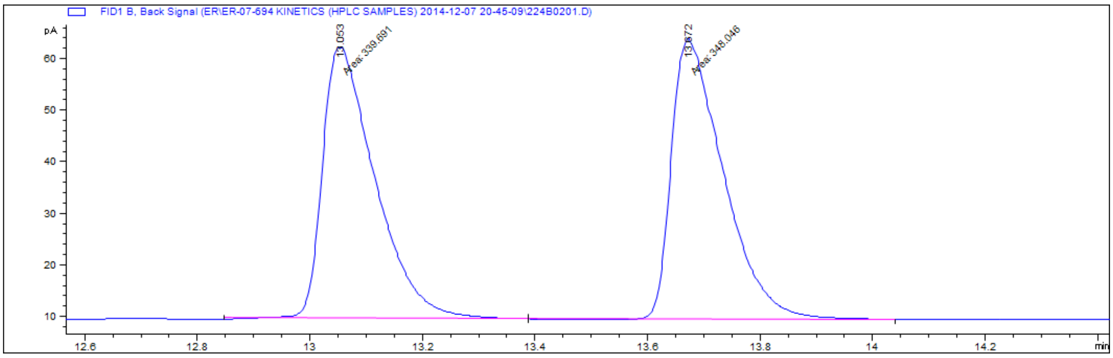

30 min

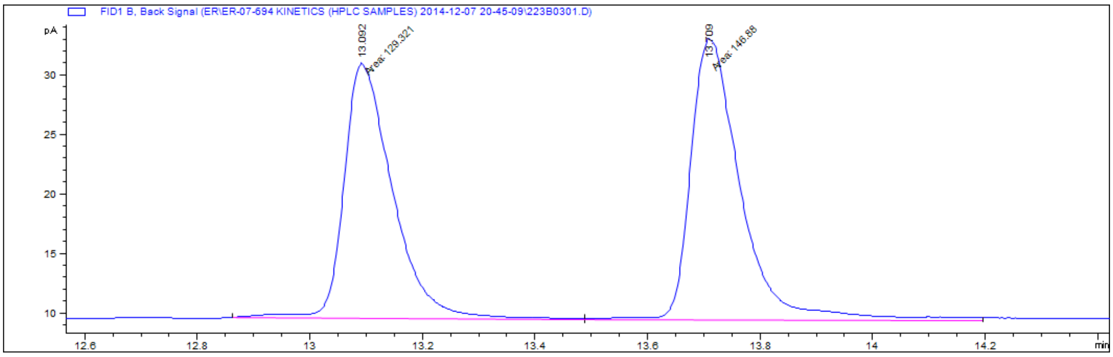

1 h

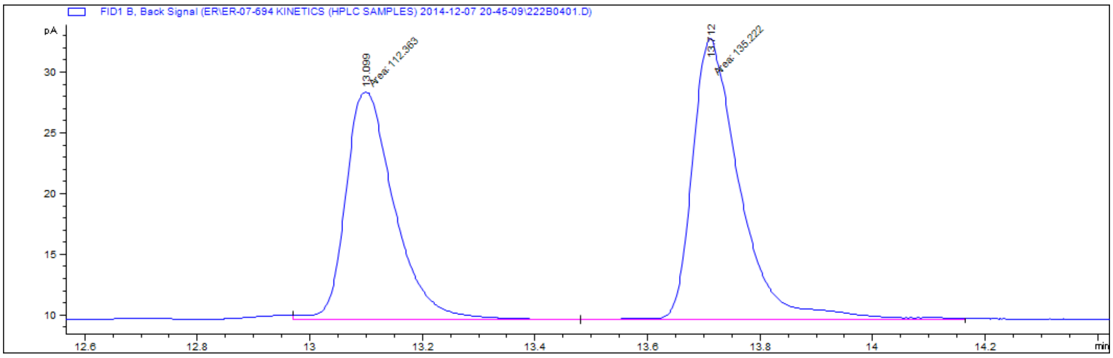

2 h

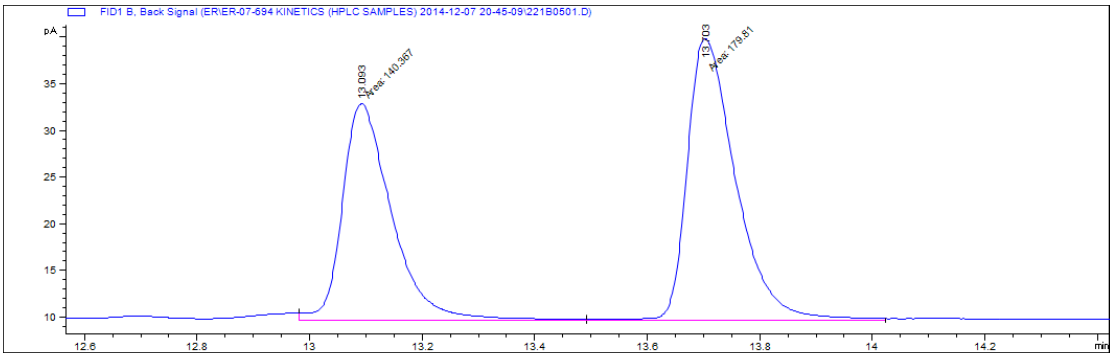

3 h

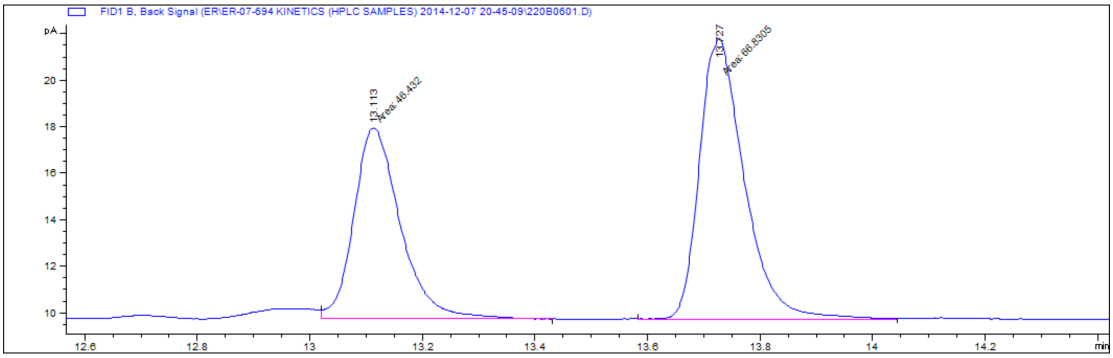

Overnight

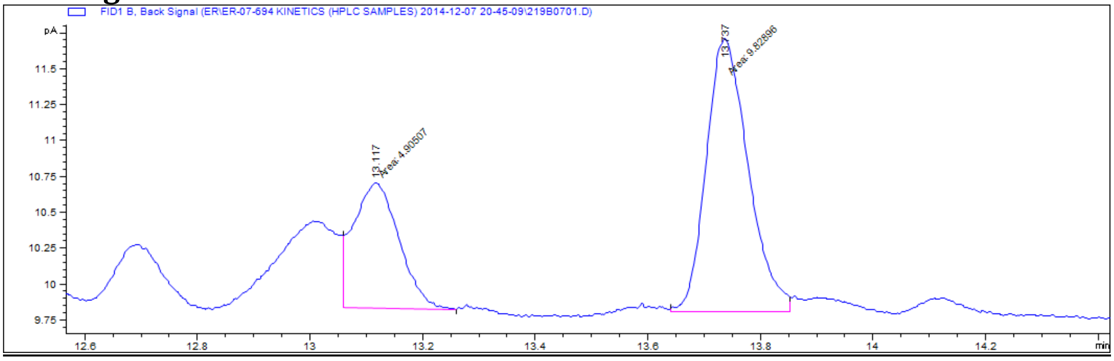

Racemic

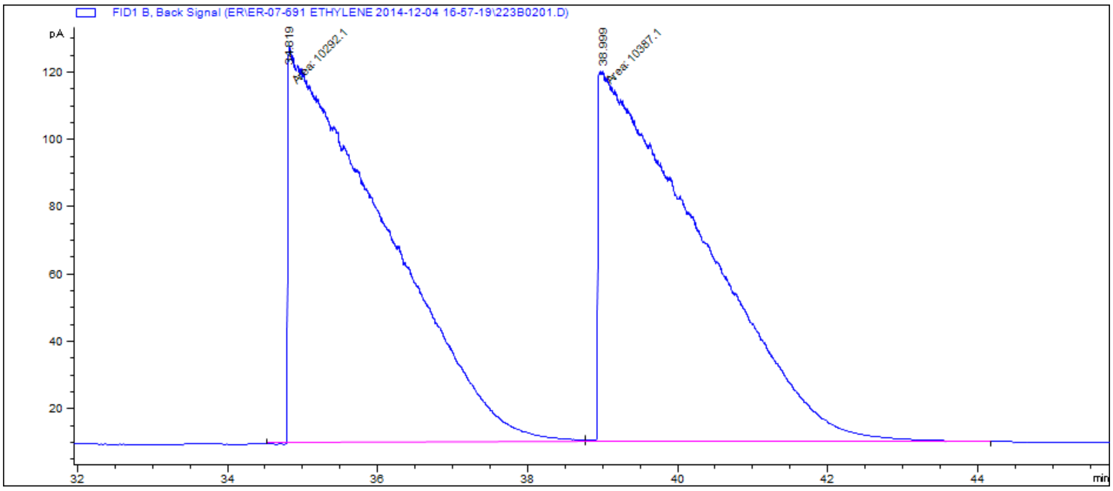

HPLC traces of 5

The enantiomeric excess of the product **4** was determined by HPLC [Chiralpak® IB; hexane:*i*PrOH 99.2:0.8; 1.0 ml.min^{-1}, λ= 210 nm, t_R = 5.67 min (minor enantiomer), t_R = 6.07 min (major enantiomer)].

5 min

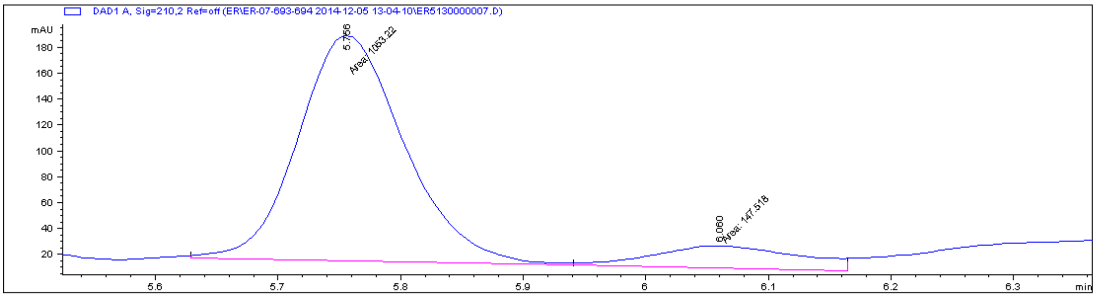

30 min

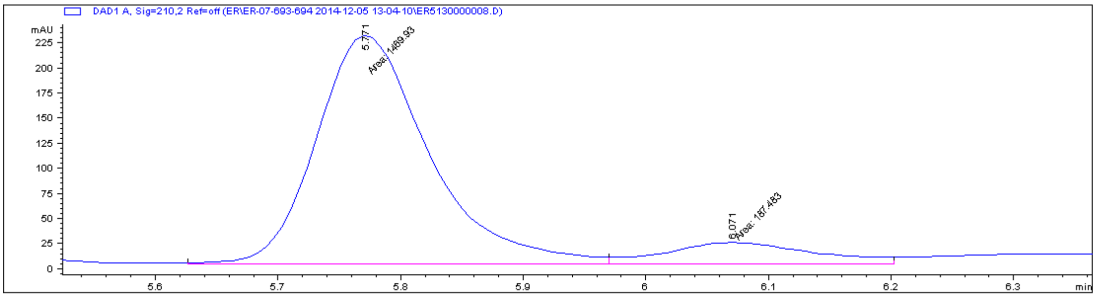

2 h

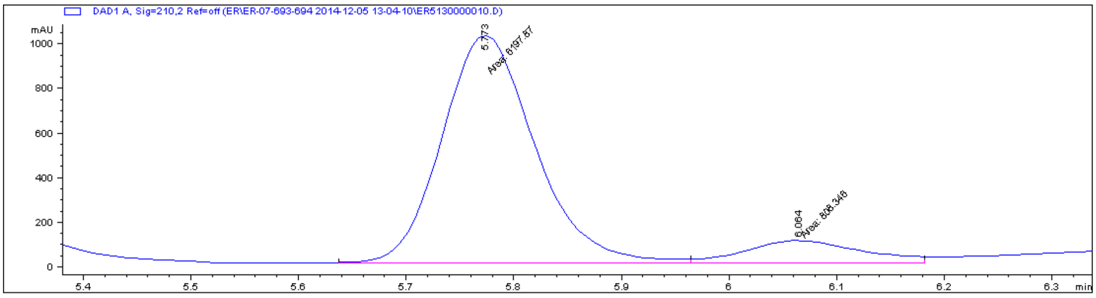

3 h

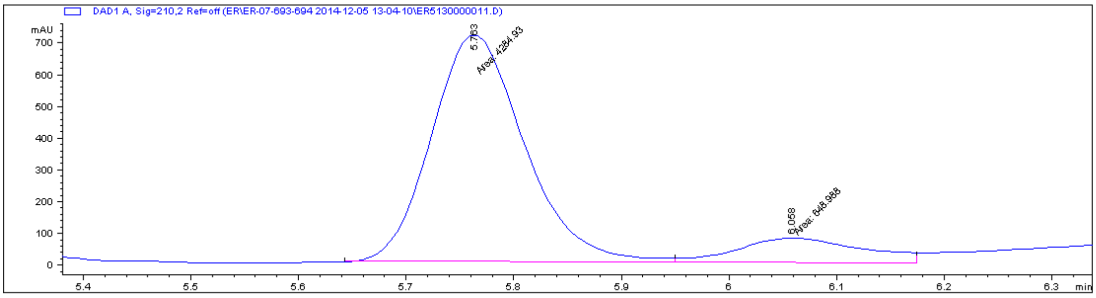

Overnight

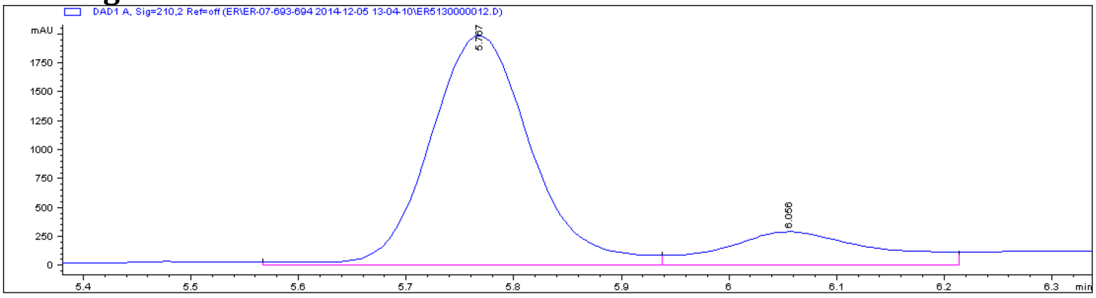

Racemic

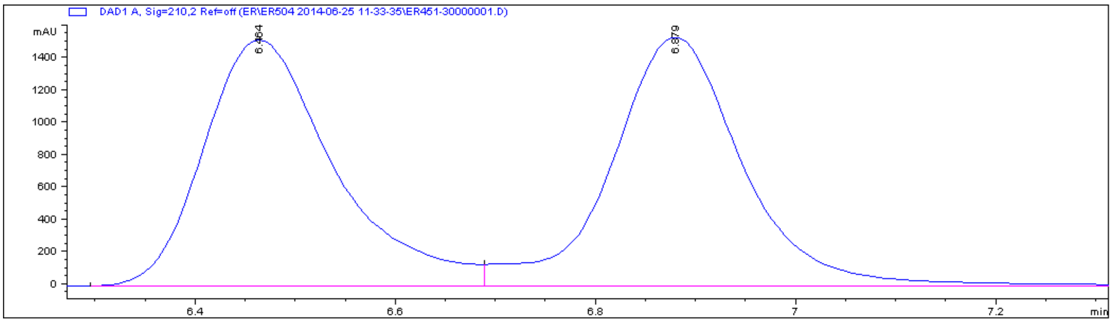

3,6-dihydro-2*H*-pyran-3-yl diethyl phosphate (2d)
Kinetic NMR

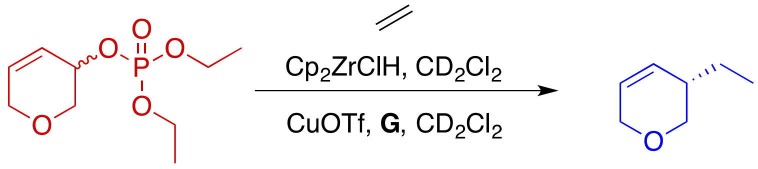

In a flame-dried flask under inert atmosphere, ethylene gas (1 atm) was bubble through a solution of Cp_2ZrHCl (103 mg, 0.4 mmol, 2.0 equiv) in CD_2Cl_2 (0.4 mL) under an argon atmosphere and stirred vigorously until a clear yellow solution was obtained (15 min). Simultaneously, in another flask under inert atmosphere, CuCl (1.9 mg, 0.02 mmol, 0.1 equiv) and (*S, S*)-**A** (9.9 mg, 0.02 mmol, 0.1 equiv) were dissolved in CD_2Cl_2 (1.0 mL) and stirred for 1 h at room temperature. AgOTf (5.7 mg, 0.022 mmol, 0.11 equiv) was added to the freshly formed Cu-ligand complex solution and stirred for 15 min. The resulting catalyst complex mixture was filtered into a flamed dried NMR tube adapted with an NMR septa. Then the freshly prepared alkylzirconocene species was added to the NMR tube and the tube was thoroughly shaken to obtain a homogenous black solution. 3,6-dihydro-2*H*-pyran-3-yl diethyl phosphate **2d** (27 μL, 0.2 mmol, 1.0 equiv) was added via a microsyringe to the NMR tube. The reaction mixture was shaken vigorously (upside-down mixing) and NMRs were recorded at regular intervals.

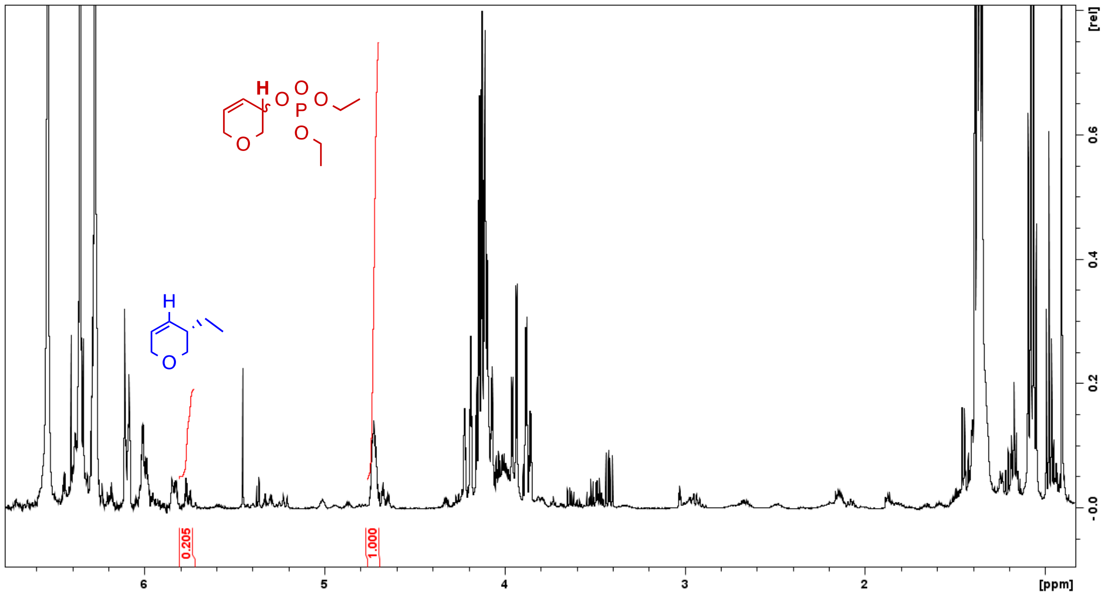

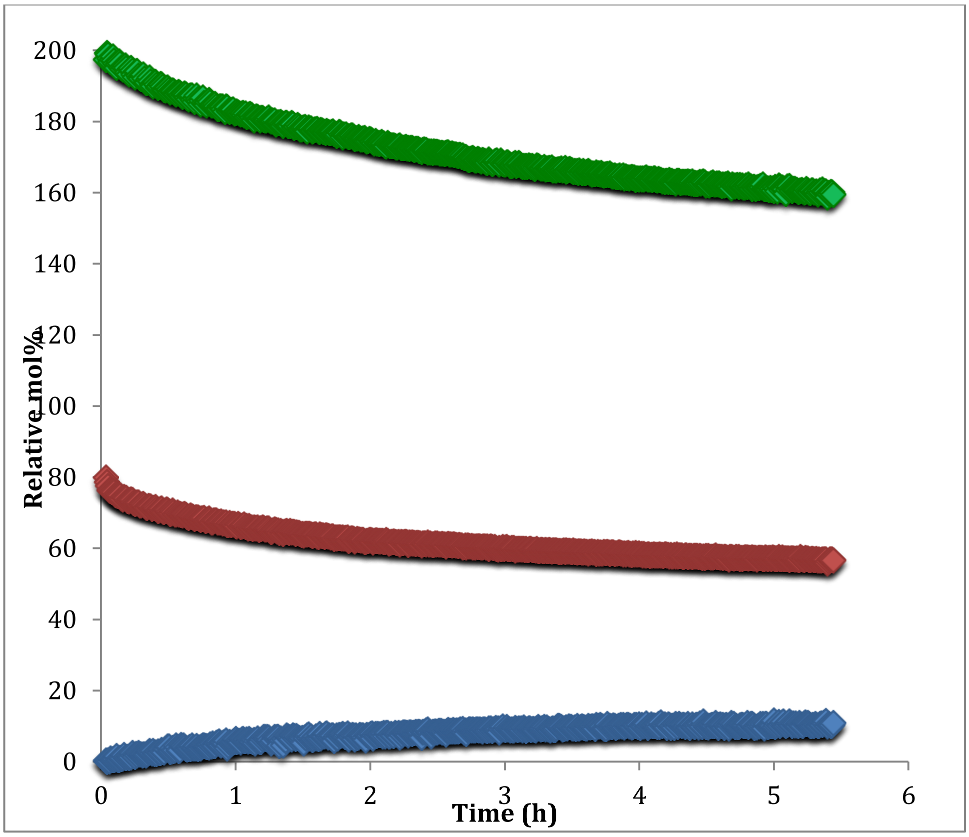

Kinetic ee

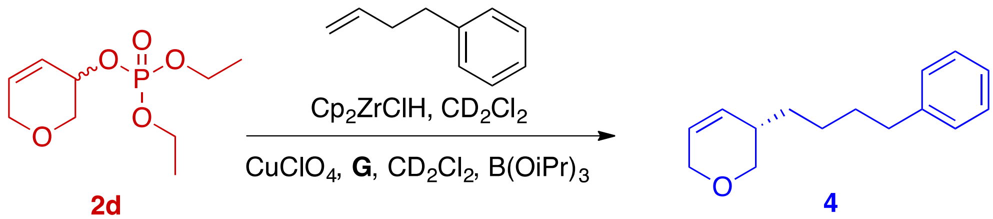

In a flame-dried flask under inert atmosphere, Cp_2ZrHCl (309 mg, 1.2 mmol, 2.0 equiv) was added to a solution of 4-phenyl-1-butene (230 μL, 1.5 mmol, 2.5 equiv) in CH_2Cl_2 (0.6 mL) under an argon atmosphere and stirred vigorously until a clear yellow solution was obtained (20–40 min). Simultaneously, in another flask under inert atmosphere, CuCl (5.7 mg, 0.06 mmol, 0.1 equiv) and (*S, S*)-**A** (29.7 mg, 0.06 mmol, 0.1 equiv) were dissolved in CH_2Cl_2 (3.0 mL) and stirred for 1 h at room temperature. AgOTf (17.1 mg, 0.066 mmol, 0.11 equiv) was added to the freshly formed Cu-ligand complex solution and stirred for 15 min. The resulting catalyst complex mixture was filtered into the freshly prepared alkylzirconocene species. After 10 min, 3,6-dihydro-2*H*-pyran-3-yl diethyl phosphate **2d** (140 mg, 0.6 mmol, 1.0 equiv) was added dropwise via a microsyringe to the resulting black solution and stirred overnight. Aliquots were taken regularly and analysed by HPLC to obtain the ee of product **5**.

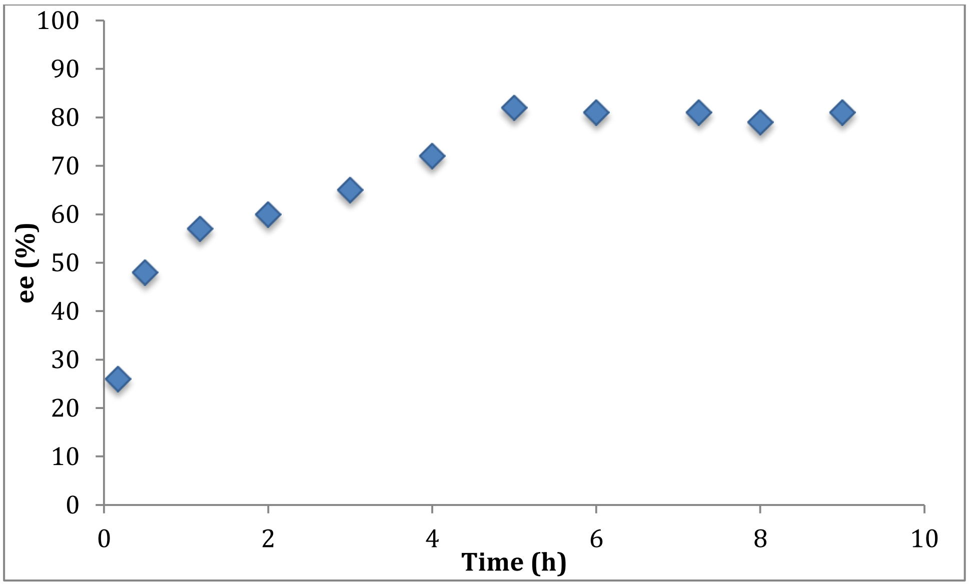

HPLC traces of 5

The enantiomeric excess of the product was determined by HPLC [Chiralpak® IB; hexane:*i*PrOH 99.2:0.8; 1.0 ml.min^{-1}, λ= 210 nm, t_R = 5.67 min (minor enantiomer), t_R = 6.07 min (major enantiomer)].

10 min

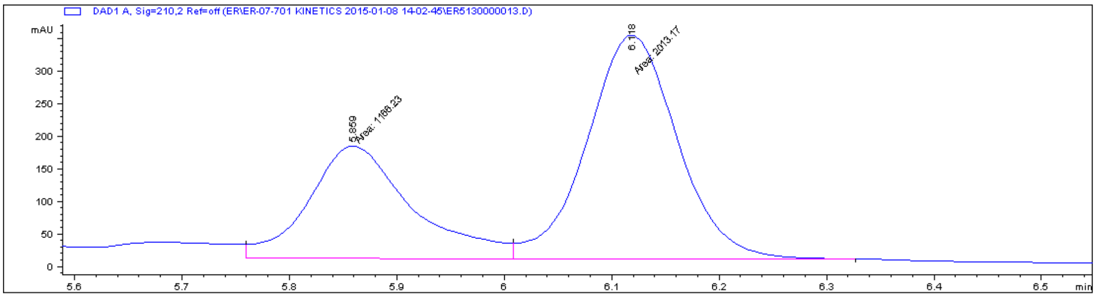

30 min

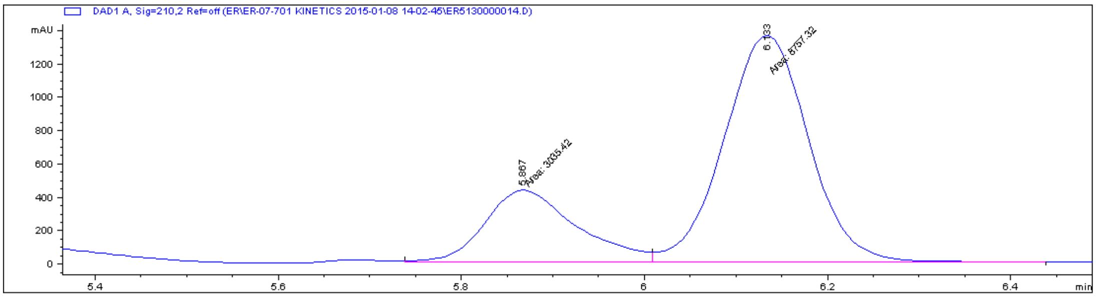

1.2 h

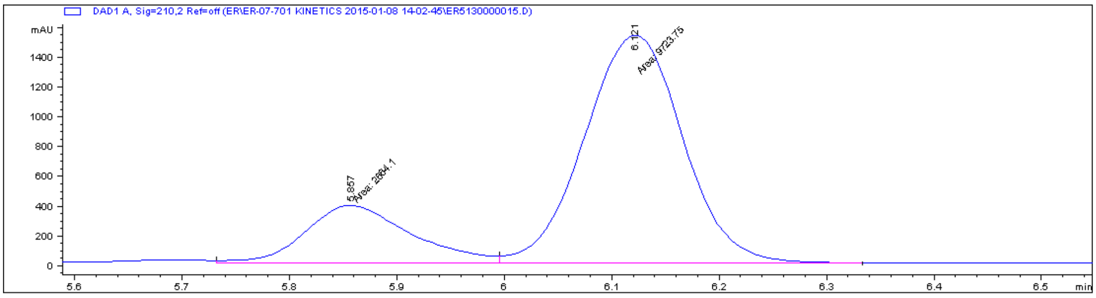

2 h

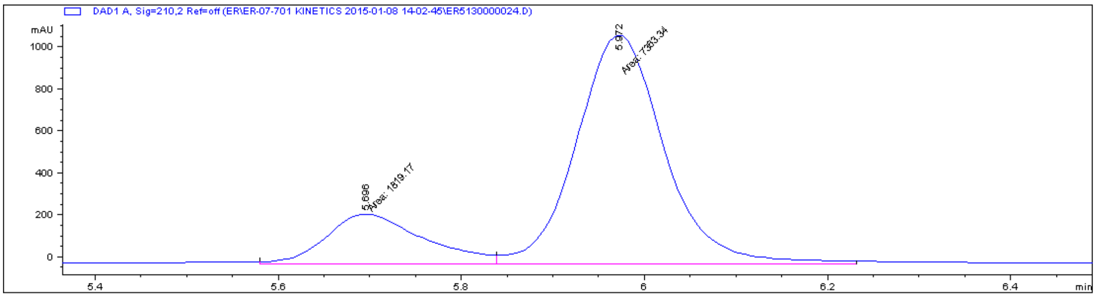

3 h

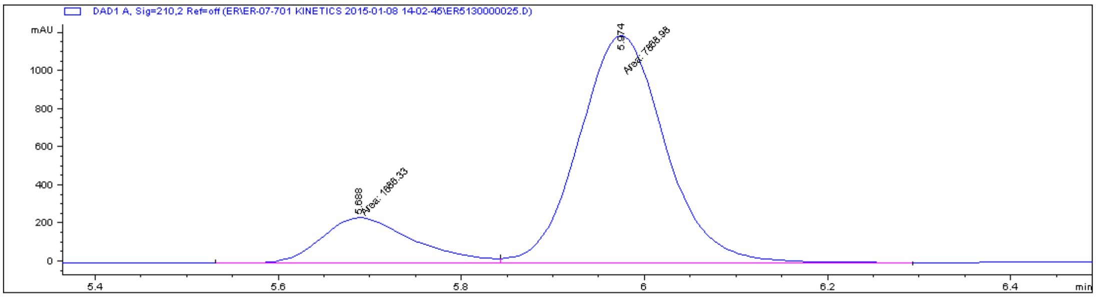

5 h

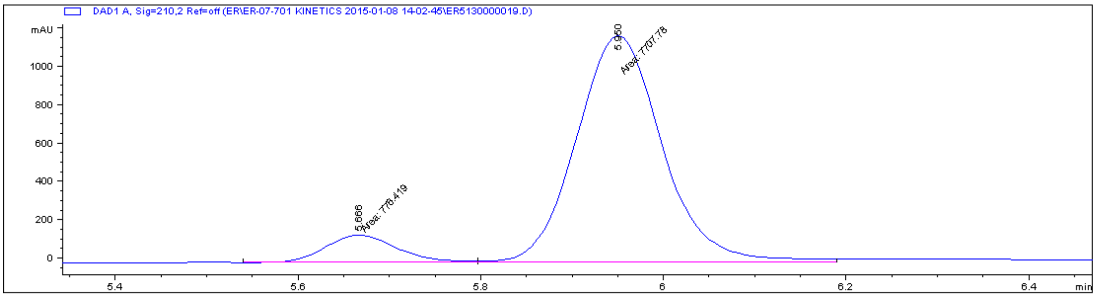

6 h

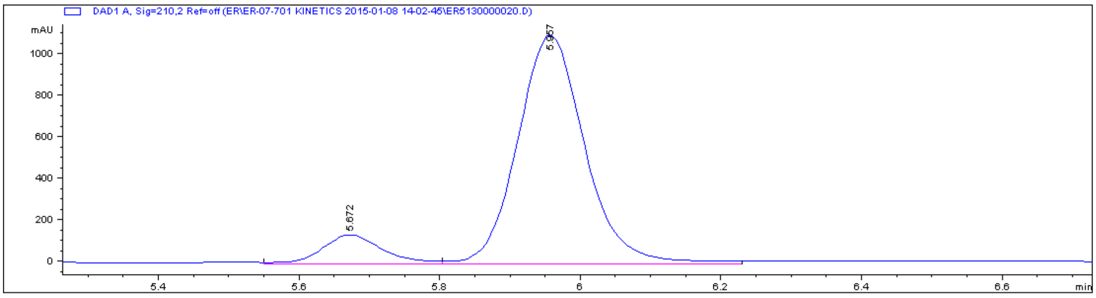

7 h

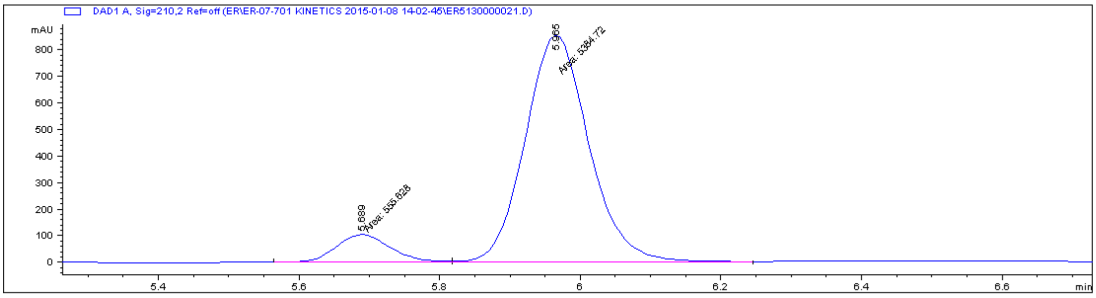

8 h

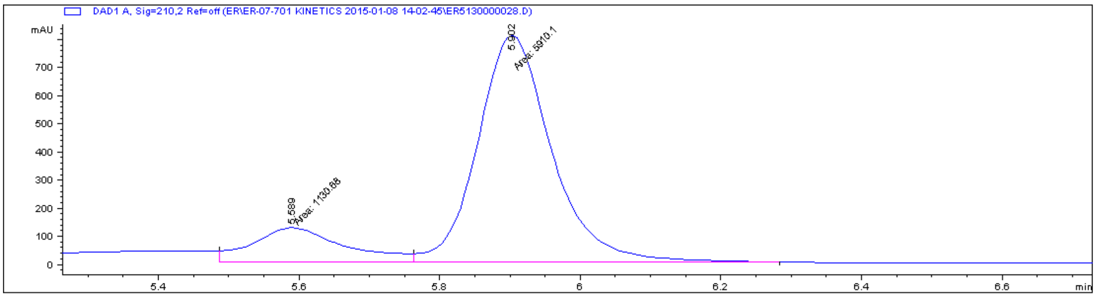

9 h

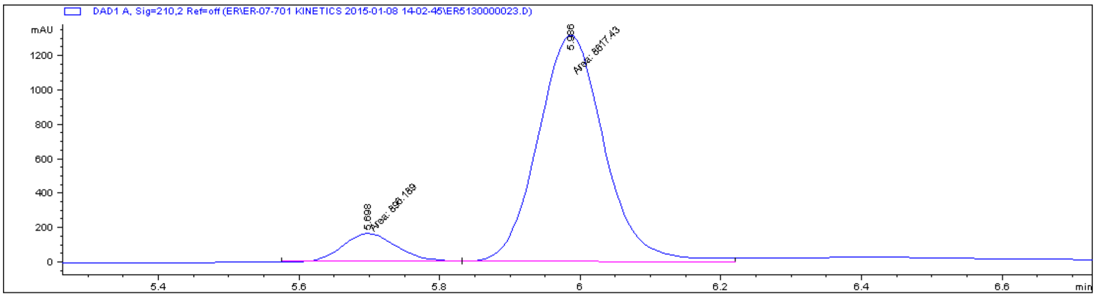

Racemic

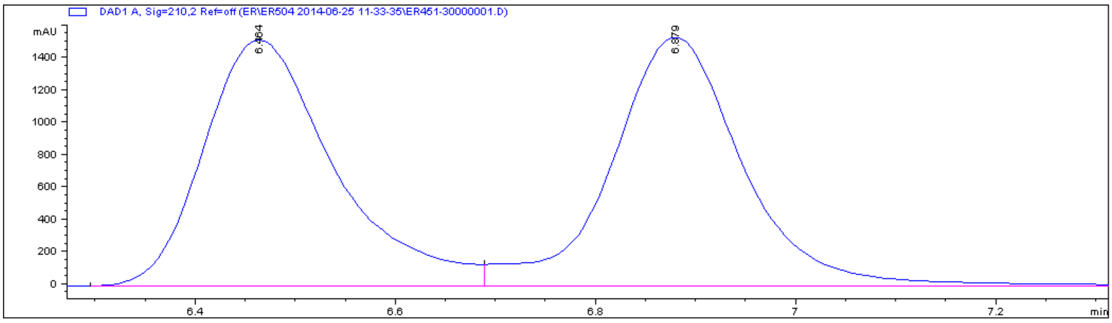

VI. References

[1] S. J. L. S. L. Buchwald, R. B. Nielsen, B. T. Watson, and S. M. King., *Org. Synth.* **1993**, *71*, 77-82.

[2] R. G. Salomon, J. K. Kochi, *J Am Chem Soc* **1973**, *95*, 3300-3310.

[3] P. M. C. Roth, University of Oxford **2014**.

[4] M. H. Katcher, A. G. Doyle, *J Am Chem Soc* **2010**, *132*, 17402-17404.

[5] Y. Onishi, Y. Nishimoto, M. Yasuda, A. Baba, *Org Lett* **2011**, *13*, 2762-2765.

[6] H. Grugel, T. Minuth, M. M. K. Boysen, *Synthesis-Stuttgart* **2010**, 3248-3258.
[7] K. Yasui, K. Fugami, S. Tanaka, Y. Tamaru, *J Org Chem* **1995**, *60*, 1365-1380.
